# Impaired skeletal muscle hypertrophy signaling and amino acid deprivation response in Apoe knockout mice with an unhealthy lipoprotein distribution

**DOI:** 10.1038/s41598-021-96000-8

**Published:** 2021-08-12

**Authors:** Jakob Agergaard, Mie Cecilie Faber Zillmer, Josué L. Castro-Mejía, Kenneth Mertz, Witold Kot, Grith Højfeldt, Gerrit van Hall, Dennis S. Nielsen, Peter Schjerling, Lars Holm

**Affiliations:** 1 Institute of Sports Medicine Copenhagen, Department of Orthopedic Surgery, Copenhagen University Hospital - Bispebjerg and Frederiksberg, Copenhagen, Denmark; 2 Department of Clinical Medicine, Center for Healthy Aging, University of Copenhagen, Copenhagen, Denmark; 3 Department of Food Science, Faculty of Science, University of Copenhagen, Copenhagen, Denmark; 4 Department of Plant and Environmental Sciences, Faculty of Science, University of Copenhagen, Copenhagen, Denmark; 5 Clinical Metabolomics Core Facility, Clinical Biochemistry, Rigshospitalet, Copenhagen, Denmark; 6 Department of Biomedical Sciences, Faculty of Health and Medical Sciences, University of Copenhagen, Copenhagen, Denmark; 7 School of Sport, Exercise and Rehabilitation Sciences, University of Birmingham, Birmingham, UK

**Keywords:** Reverse transcription polymerase chain reaction, Ageing, Metabolism, Cell biology

## Abstract

This study explores if unhealthy lipoprotein distribution (LPD) impairs the anabolic and amino acid sensing responses to whey-protein feeding. Thus, if impairment of such anabolic response to protein consumption is seen by the LPD this may negatively affect the skeletal muscle mass. Muscle protein synthesis (MPS) was measured by puromycin labeling in Apolipoprotein E knockout (Apoe KO), characterized by an unhealthy LPD, and wild type mice post-absorptive at 10 and 20 weeks, and post-prandial after whey-protein feeding at 20 weeks. Hypertrophy signaling and amino acid sensing mechanisms were studied and gut microbiome diversity explored. Surprisingly, whey-protein feeding did not affect MPS. p-mTOR and p-4E-BP1 was increased 2 h after whey-protein feeding in both genotypes, but with general lower levels in Apoe KO compared to wild type. At 20 weeks of age, Apoe KO had a greater mRNA-expression for SNAT2, CD98, ATF4 and GCN2 compared to wild type. These responses were not associated with gut microbiota compositional differences. Regardless of LPD status, MPS was similar in Apoe KO and wild type. Surprisingly, whey-protein did not stimulate MPS. However, Apoe KO had lower levels of hypertrophy signaling, was amino acid deprived, and had impaired amino acid sensing mechanisms.

## Introduction

A demographic change towards an increase in the elderly population is causing an increase in the socioeconomic demand for care of the elderly population. Notably, a detrimental loss of skeletal muscle mass and function is occurring as we age^1^, which may compromise independence as well as metabolic health. The cause of this muscle deterioration is multifactorial^2^, but especially the ability of skeletal muscle to respond to anabolic stimuli such as exercise, nutrition and growth factors are compromised^3–6^.

Anabolic resistance is seen as a reduced anabolic response of the skeletal muscle towards circulating amino acids, e.g. in the postprandial phase following a protein supplement or meal. Under basal conditions, the rates of protein turnover in elderly individuals are not abnormal^7^, but the ability to stimulate muscle protein synthesis is impaired in elderly compared to young^8^ following protein or amino acid (AA) ingestion^3,9^. Furthermore, it takes more protein to maximally stimulate postprandial rates of protein synthesis in elderly compared to young individuals^10^. However, data from other experiments is inconsistent as studies have also shown no impaired protein synthesis response in elderly^11,12^.

The essential amino acid (EAA) leucine is the most potent stimulator of muscle protein synthesis^13^. Thus, dietary protein rich in leucine and EAAs to serve as building blocks, are important with regards to stimulating protein synthesis. Whey protein meets this criteria by being rich in EAAs, especially leucine, and is in addition highly digestible and fast absorbed underpinning its potency for stimulating muscle protein synthesis^14,15^.

Protein synthesis is mediated by signaling through the mechanistic target of rapamycin complex 1 (mTORC1). Amino acids sensing and transport across the plasma membrane by L-type amino acid transporter 1 (LAT1)/solute-linked carrier (SLC) 7A5 and sodium-coupled neutral amino acid transporter 2 (SNAT2)/SLC38A2^16,17^ is orchestrating a series of downstream signaling events. These signals activate the Rag proteins that initiate formation of sites at the lysosome for mTORC1 docking and activation^18,19^. In response to amino acid deprivation, the mTORC1 inhibitor General Control Nonderepressible 2 (GCN2) is activated. GCN2 phosphorylates translation initiation factor 2 alpha (eIF2α) in response to amino acid starvation, which inhibits general protein synthesis and induces expression of activating transcription factor 4 (ATF4), a transcriptional activator of the general amino acid control pathway^20^. Starvation induced increase in ATF4 expression leads to SNAT2 and LAT1 upregulation, as ATF4 serves as transcription factor for SNAT2 and LAT1^21,22^.

With increasing age, a change in the plasma lipid profile is seen towards a more atherogenic profile with increased triglyceride, cholesterol and low density lipoproteins (LDL) levels and lower high density lipoproteins (HDL) levels^23–25^. Such an altered lipid profile and unhealthy lipoprotein distribution (LPD) could be one factor underlying the sarcopenic process. Hida and colleagues have shown that persons with sarcopenia have an unhealthy lipoprotein distribution seen as higher total cholesterol and LDL levels compared to persons with normal muscle mass and function^26^. Furthermore, Silveira and colleagues found that hand grip strength was negatively associated with hypercholesterolemia^27^. The underlying effect of the sarcopenic development could be an induction of anabolic resistance, but currently, no clear causation of the relationship to an unhealthy LPD exists. At this point it has been shown that basal protein synthesis is lower in rats with elevated plasma free fatty acids^28^. Moreover, lipid infusion has been shown to block the muscle protein synthesis response to an intake of amino acids^29^. Opposing observations have been made by others^30^. To our knowledge, nobody has directly investigated the effect of an unhealthy lipoprotein distribution (LPD) on the signaling to muscle protein synthesis. High levels of LDL, low levels of HDL and high levels of triglycerides in plasma characterize an unhealthy LPD. Interestingly, the gut microbiome (GM) contributes to a substantial proportion of the variation in circulating blood lipids including lipoproteins^31^. In older adults physical fitness is linked to alterations in both circulating blood lipids/lipoproteins and gut microbiome signatures^32^. The aim of the current study was to investigate how a characteristic of aging, the unhealthy lipoprotein distribution, influences the sensitivity towards elevated levels of circulating amino acids; specifically the study applied a bolus of whey protein feeding in 20 week old Apoe knockout (KO) mice with an unhealthy LPD compared to WT control mice. Furthermore, signaling implicated in protein synthesis and mRNA expression of targets implicated in amino acid sensing as well as the gut microbiome were investigated. Factors disturbing the hypertrophy signaling pathway, and especially the ability of these pathways to respond to hyperaminoacidemia, could have an effect on the anabolic resistance. We hypothesized that an unhealthy LPD obtained in Apoe KO mice is associated with a diminished muscle protein synthesis rate and intrinsic mTORC1 signaling response from basal, fasted state to hyperaminoacidemia after protein feeding.

## Results

### Mouse characteristics

**Figure 1:**
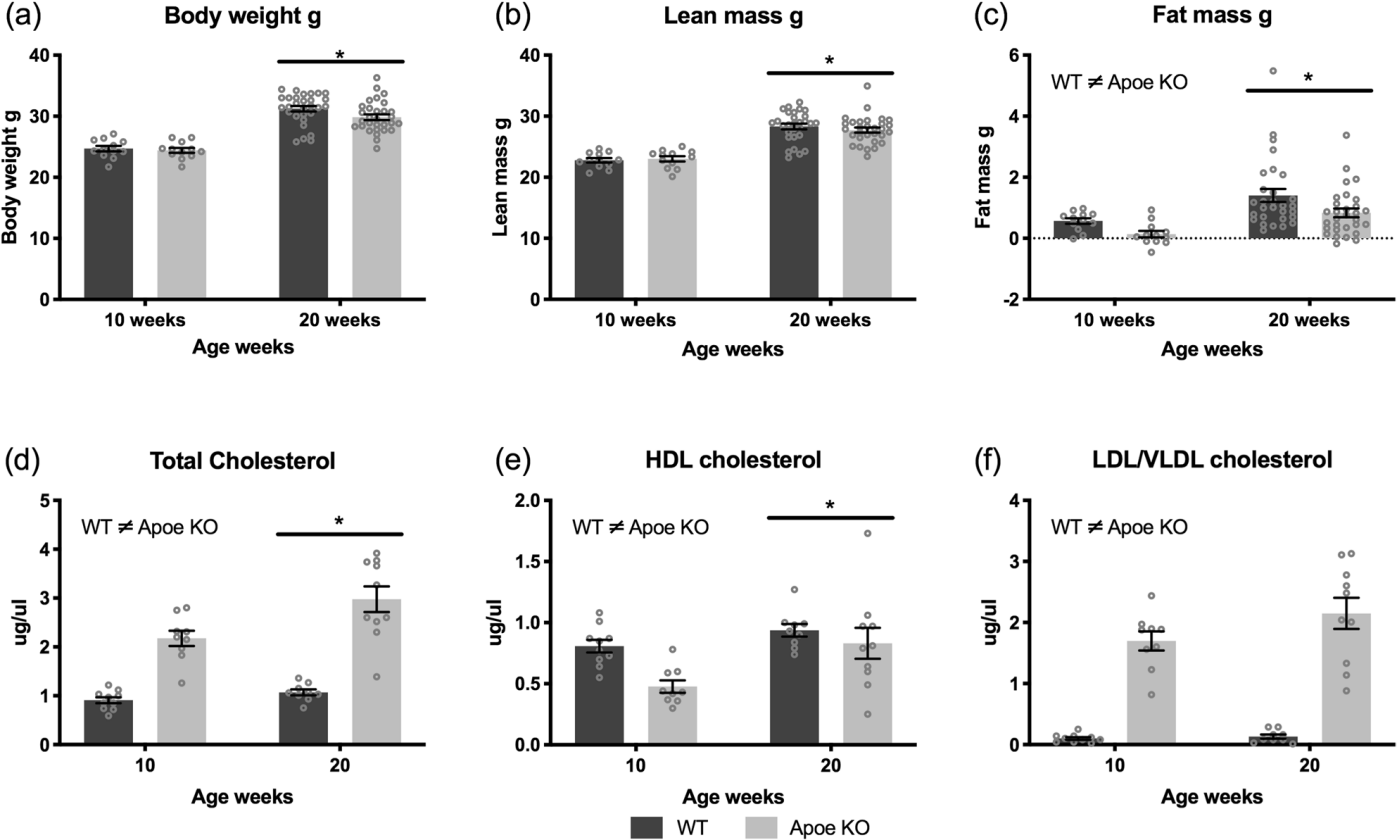
Mouse characteristics. WT (dark bars) and Apoe KO (grey bars) body weight (**A**), lean body mass (**B**), fat mass (**C**), total cholesterol (HDL + LDL/VLDL) (**D**), HDL cholesterol (**E**) and LDL/VLDL cholesterol (**F**) at 10 and 20 weeks. For (**A**–**C**); WT n = 12 at 10 weeks and n = 29 at 20 weeks, Apoe KO n = 12 at 10 weeks and n = 30 at 20 weeks. For D, E and F; WT n = 10 at 10 weeks and n = 9 at 20 weeks, Apoe KO n = 9 at 10 weeks and n = 9 at 20 weeks. Individual values are shown with bars indicating means ± SEM, *difference between time points, *p* < 0.05.

The mice were characterized at 10 and 20 weeks of age in the fasted state. Both genotypes increased their weight and LBM from 10 to 20 weeks of age (both *p* < 0.001) (Fig. 1A,B). Apoe KO mice had significantly lower fat mass compared to WT (*p* = 0.017), but both genotypes increased fat mass from 10 to 20 weeks of age (*p* < 0.001) (Fig. 1C).

HDL cholesterol, LDL/VLDL cholesterol and total cholesterol concentrations were measured at 10 and 20 weeks to confirm the phenotype of the Apoe KO mice. Apoe KO mice had higher total cholesterol levels compared to WT (*p* < 0.001) and total cholesterol increased in both genotypes from 10 to 20 weeks (*p* = 0.006) (Fig. 1D). WT mice had higher HDL cholesterol concentration than Apoe KO mice (*p* < 0.001) and the HDL cholesterol concentration increased in both genotypes from 10 to 20 weeks (*p* = 0.008) (Fig. 1E). Furthermore, Apoe KO mice had significantly higher LDL/VLDL cholesterol concentration compared to WT mice at all time points (*p* < 0.001) (Fig. 1F).

### 10 to 20 weeks of age comparison

**Figure 2:**
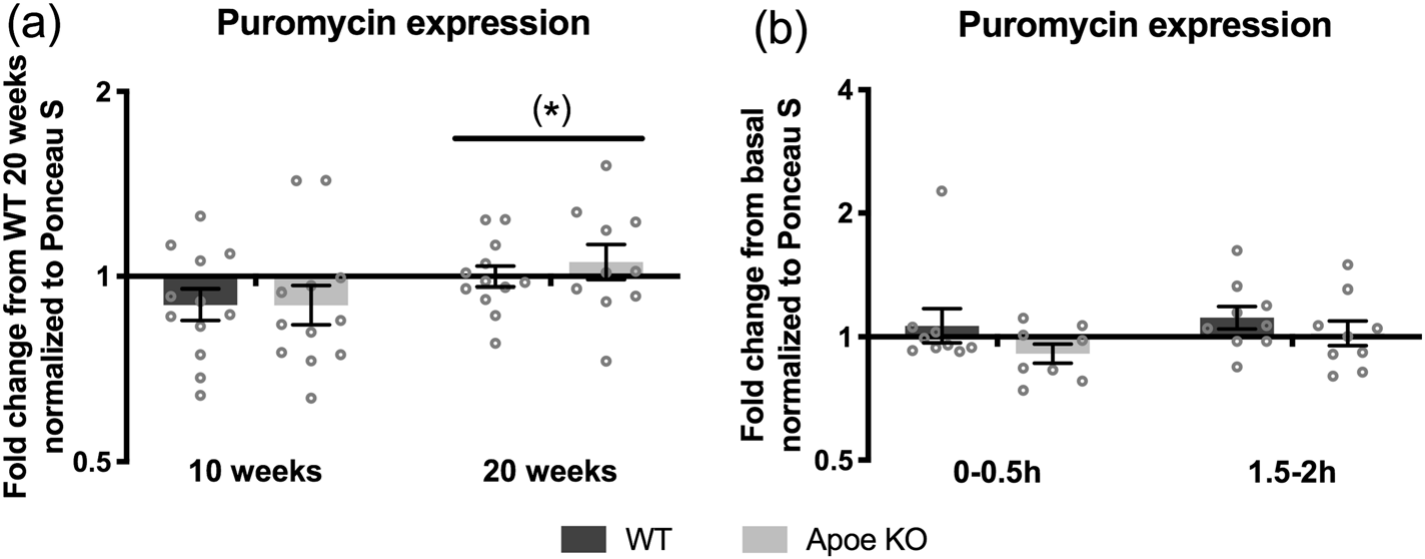
Puromycin incorporation in WT (dark bars) and Apoe KO (grey bars) mice at 10 (WT n = 12, Apoe KO n = 12) and 20 weeks (WT n = 12, Apoe KO n = 10) of age (**A**) and at 0–0.5 h (WT n = 9, Apoe KO n = 9) and 1.5-2 h (WT n = 9, Apoe KO n = 9) after whey protein feeding (**B**). Data were normalized to Ponceau S and in (**A**) expressed as fold change from WT fasted at 20 weeks of age and in (**B**) expressed as fold change from basal. Individual values are shown with bars indicating geometric means ± back-transformed SEM. (*) trend for a difference between time points 0.05 ≤ *p* < 0.10. Full lane representative blots are shown in Supplementary Figure S1.

Mixed muscle protein synthesis (MPS) was measured in WT and Apoe KO mice through puromycin incorporation over a period of 30 min^15,33,34^. Full lane representative blots are shown in Supplemental Figure S1. In general, at 10 and 20 weeks in the fasted state there was no difference in puromycin incorporation between WT and Apoe KO mice. A trend was seen towards a greater basal MPS at 20 weeks compared to 10 weeks of age *p* = 0.069 (Fig. 2A).

**Figure 3:**
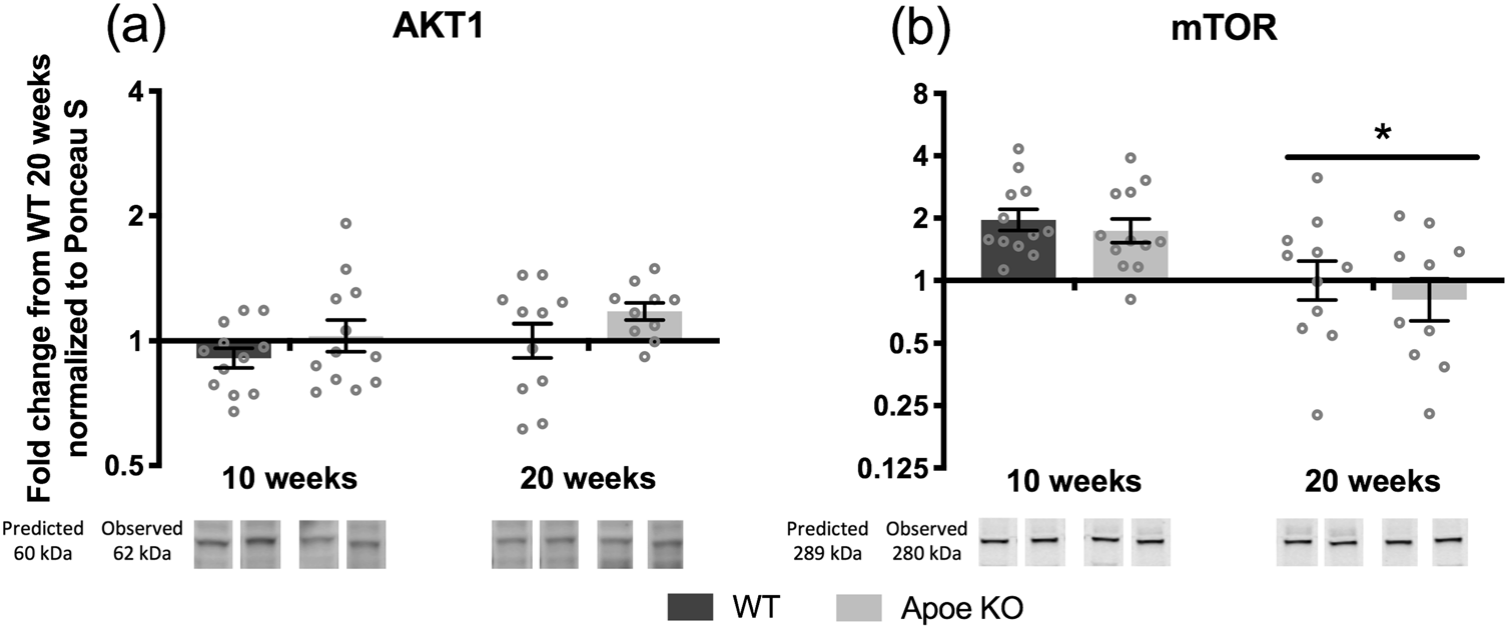
Hypertrophy signaling pathway at 10 (WT n = 12, Apoe KO n = 12) and 20 (WT n = 12, Apoe KO n = 10) weeks fasted. Protein expression of AKT1 (**A**), mTOR (**B**), in WT (dark bars) and Apoe KO (grey bars) mice at 10 and 20 weeks of age. Data were normalized to Ponceau S and expressed as fold change from WT 20 weeks and individual values are shown with bars indicating geometric means ± back-transformed SEM. * difference between time points, *p* < 0.05. Samples were loaded in a randomized order, repeated twice on the same gel. Therefore, representative images of the bands are cropped and reorganized to represent the respective time point and mouse. Expected and observed band size is indicated for each target.

In the fasted state at 10 and 20 weeks of age, total protein levels of AKT1 was measured with no changes at any time point (Fig. 3A), whereas total mTOR decreased from 10 to 20 weeks of age (*p* < 0.001) (Fig. 3B). There were no significant differences between WT and Apoe KO mice in these targets.

**Figure 4:**
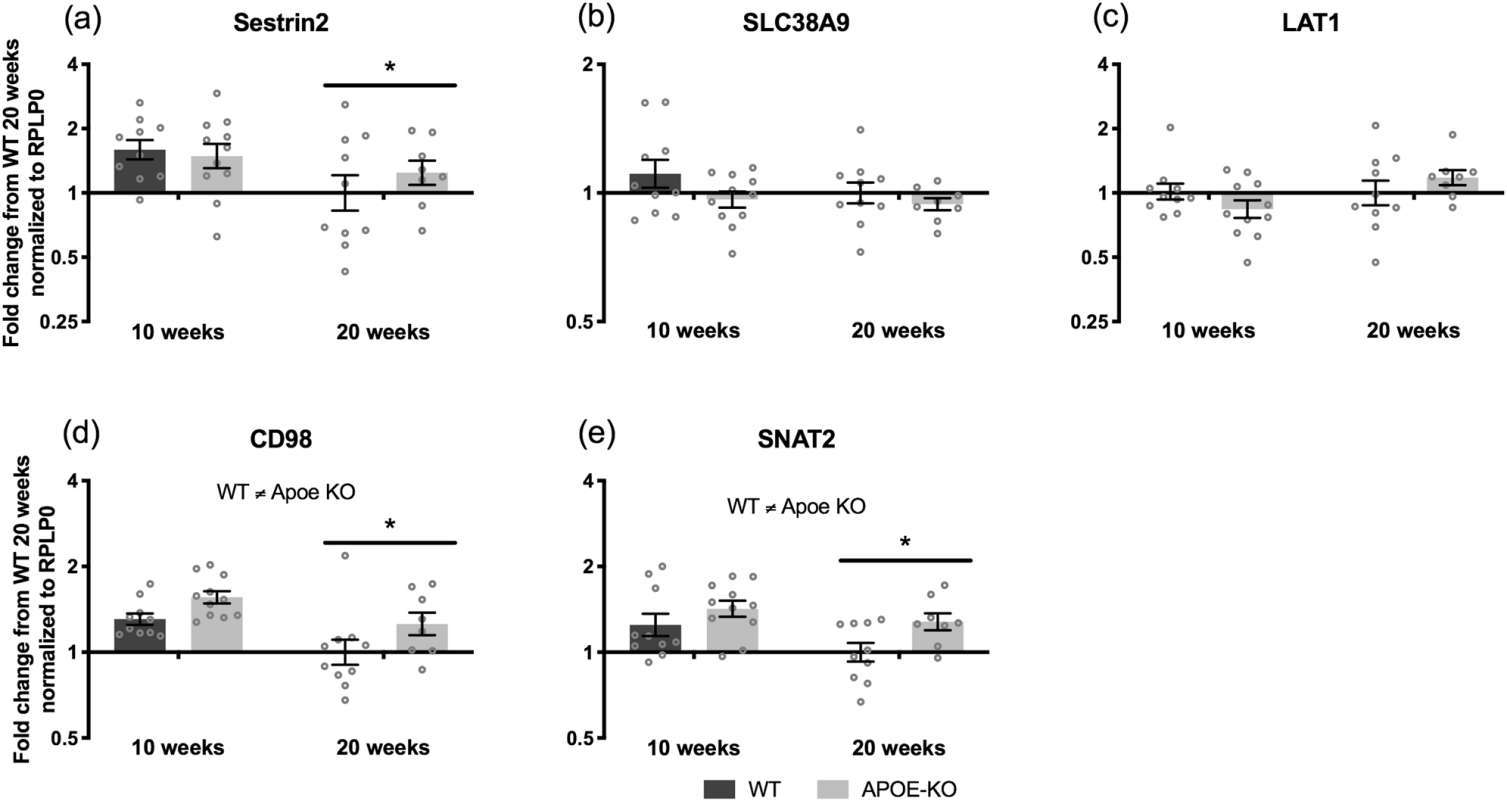
Amino acid transporters and sensors at 10 (WT n = 10, Apoe KO n = 11) and 20 (WT n = 10, Apoe KO n = 8) weeks fasted. mRNA expression in WT (dark bars) and Apoe KO (grey bars) mice of Sestrin 2 (**A**), SLC38A9 (**B**), LAT1 (**C**), CD98 (**D**), and SNAT2 (**E**) at 10 and 20 weeks. Data were normalized to RPLP0 and expressed as fold change from WT 20 weeks and individual values are shown with bars indicating geometric means ± back-transformed SEM. * difference between time points, *p* < 0.05.

For the amino acid transporters and sensors an overall decrease in the intracellular leucine sensor Sestrin2 mRNA expression from 10 to 20 weeks was found (*p* = 0.033) (Fig. 4A). mRNA expression of the lysosomal arginine sensor SLC38A9 did not change significantly from 10 to 20 weeks, but there was a trend for Apoe KO mice having lower expression than WT mice (*p* = 0.083) (Fig. 4B). A trend for an interaction (genotype × age) (*p* = 0.096) was found for the amino acid transporter LAT1 (Fig. 4C). The mRNA expression of the LAT1 associated glycoprotein CD98 was significant different between WT and Apoe KO mice with CD98 mRNA being higher in the Apoe KO mice (*p* = 0.010) (Fig. 4D). Furthermore, overall CD98 mRNA expression decreased from 10 to 20 weeks of age (*p* = 0.003) (Fig. 4D). From 10 to 20 weeks of age, overall SNAT2 mRNA decreased (*p* = 0.039) and SNAT2 mRNA expression was significantly higher in Apoe KO than WT (*p* = 0.019) (Fig. 4E).

**Figure 5:**
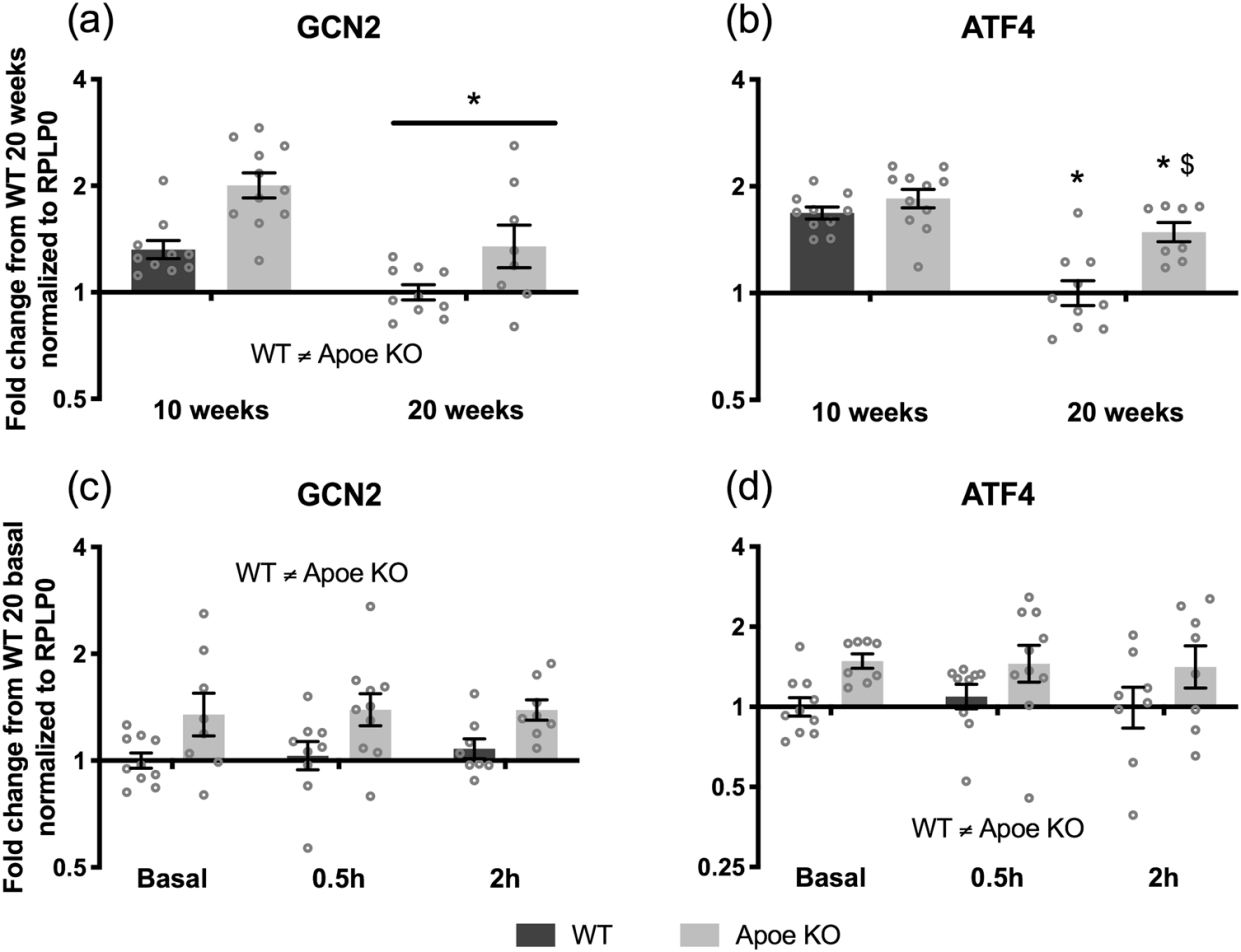
Amino acid deprivation response pathway. mRNA expression of GCN2 (A + C), ATF4 (B + D), in WT (dark bars) and Apoe KO (grey bars) at 10 weeks (WT n = 10, Apoe KO n = 11) and 20 weeks (WT n = 10, Apoe KO n = 8) basal, 0.5 h (WT n = 9, Apoe KO n = 9), and 2 h (WT n = 8, Apoe KO n = 8) after whey-protein ingestion. Data were normalized to RPLP0 and expressed as fold change from WT 20 weeks/basal and individual values are shown with bars indicating geometric means ± back-transformed SEM. * difference between time points *p* < 0.05, $ different from WT mice *p* < 0.05.

mRNAs of targets implicated in the amino acid deprivation response were also investigated using RT-qPCR. GCN2 mRNA levels decreased from 10 to 20 weeks (*p* < 0.001) and GCN2 mRNA expression were in general higher in Apoe KO mice (*p* < 0.001) (Fig. 5A). Furthermore, an interaction between time and genotype was found for ATF4, where Apoe KO and WT ATF4 mRNA expression decreased from 10 to 20 weeks (*p* = 0.021 and *p* < 0.001, Apoe KO and WT respectively) with Apoe KO having higher ATF4 mRNA expression at 20 weeks compared to WT control (*p* < 0.001) (Fig. 5B).

### Whey protein response at 20 weeks of age

As a result of the whey protein administration the muscle free amino acid (MFAA) concentration increased over time (Table 1). The branched chain amino acid (BCAA) (*p* < 0.001), EAA (*p* < 0.001) and total AA concentrations (*p* = 0.011) were greater at 2 h compared to basal. Also, BCAA concentrations were greater at 0.5 h compared to basal (*p* = 0.009) and for EAA at 2 h compared to 0.5 h (*p* = 0.014).

**Table 1:** Muscle free AA concentrations in μM at basal and at 0.5 and 2 h post whey feeding in Apoe KO and WT mice (n = 4 at each time point of each mouse).

	Basal	0.5 h	2 h	Mice difference
**Aspartic acid**				
Apoe KO	199 ± 31	226 ± 43	296 ± 30 b,c	*p* = 0.755
WT	176 ± 42	177 ± 13	343 ± 21 b,c	
**Glutamic acid**				
Apoe KO	791 ± 115	830 ± 179	858 ± 179	***p* = 0.037**
WT	495 ± 131	472 ± 159	666 ± 146	
**Serine**				
Apoe KO	227 ± 29	297 ± 35	242 ± 10	*p* = 0.113
WT	281 ± 86	296 ± 22	415 ± 82	
**Glycine**				
Apoe KO	936 ± 100	1134 ± 178	931 ± 54	*p* = 0.309
WT	1062 ± 96	1034 ± 100	1192 ± 107	
**Asparagine**				
Apoe KO	74.8 ± 10.8	107.3 ± 17.8	97.8 ± 3.1	*p* = 0.140
WT	71.8 ± 11,4	79.0 ± 1,9	89.0 ± 9.8	
**Glutamine**				
Apoe KO	1249 ± 24	1471 ± 134	1398 ± 48	***p* = 0.047**
WT	1118 ± 146	1035 ± 112	1342 ± 145	
**Histidine**				
Apoe KO	111.0 ± 9.7	117.0 ± 2.1	115.0 ± 3.8	*p* = 0.886
WT	106.5 ± 16.4	112.0 ± 2.1	128.3 ± 16.1	
**Threonine**				
Apoe KO	191 ± 17	252 ± 30	369 ± 29 b,c	*p* = 0.773
WT	183 ± 33	251 ± 10	360 ± 20 b,c	
**Alanine**				
Apoe KO	983 ± 233	1048 ± 147	1130 ± 118	*p* = 0.153
WT	1106 ± 47	1159 ± 115	1421 ± 137	
**Proline**				
Apoe KO	223.3 ± 20.9	219.3 ± 8.5	248.0 ± 18.3	***p* = 0.028**
WT	186.0 ± 22.4	201.0 ± 9.6	213.3 ± 6.8	
**Arginine**				
Apoe KO	264 ± 67	194 ± 6	221 ± 26	***p* = 0.017**
WT	158 ± 26	154 ± 22	161 ± 36	
**Tyrosine**				
Apoe KO	60.7 ± 4.1	93.3 ± 17.4 a	63.3 ± 1.8 c	*p* = 0.093
WT	74.8 ± 12.0	116.3 ± 13.4 a	78.3 ± 11.3 c	
**Valine**				
Apoe KO	180 ± 22	265 ± 57 a	392 ± 20 b,c	*p* = 0.590
WT	159 ± 14	258 ± 14 a	378 ± 33 b,c	
**Methionine**				
Apoe KO	72.5 ± 5.5	84.5 ± 4.0 a	102.5 ± 7.2 b	*p* = 0.129
WT	66.0 ± 9.4	90.3 ± 5.5 a	78.5 ± 4.8 b	
**Isoleucine**				
Apoe KO	98 ± 8	180 ± 37 a	225 ± 31 b	*p* = 0.293
WT	85 ± 11	177 ± 12 a	179 ± 19 b	
**Leucine**				
Apoe KO	148 ± 14	281 ± 61 a	345 ± 39 b	*p* = 0.381
WT	129 ± 14	268 ± 21 a	296 ± 39 b	
**Tryptophan**				
Apoe KO	20.8 ± 1.5	28.0 ± 1.5 a	20.0 ± 0.8	*p* = 0.238
WT	20.3 ± 1.4	30.5 ± 3.1 a	26.0 ± 5.1	
**Phenylalanine**				
Apoe KO	89.3 ± 9.1	104.5 ± 6.7	87.8 ± 2.6	*p* = 0.579
WT	87.8 ± 8.7	116.8 ± 7.2	89.5 ± 14.5	
**Lysine**				
Apoe KO	532 ± 117	461 ± 22	693 ± 81 b,c	*p* = 0.487
WT	372 ± 73	446 ± 60	719 ± 128 b,c	
**BCAA**				
Apoe KO	427 ± 42	726 ± 154 a	962 ± 88 b	*p* = 0.405
WT	373 ± 39	702 ± 45 a	853 ± 82 b	
**EAA**				
Apoe KO	1449 ± 198	1772 ± 207	2349 ± 160 b,c	*p* = 0.404
WT	1209 ± 113	1748 ± 60	2253 ± 231 b,c	
**Total AA**				
Apoe KO	6316 ± 579	7391 ± 875	7833 ± 117 b	*p* = 0.489
WT	5936 ± 534	6472 ± 120	8172 ± 631 b	

(a) denotes difference between basal and 0.5 h (*p* < 0.05), b) denotes difference between basal and 2 h (*p* < 0.05), (c) denotes difference between 0.5 and 2 h (*p* < 0.05). Data shown as mean ± SEM.

At single AA level tyrosine, methionine, tryptophan, valine, isoleucine and leucine concentrations were greater at 0.5 h compared to basal (all *p* < 0.05). At 2 h compared to basal the concentrations of aspartic acid, threonine, valine, methionine, isoleucine, leucine and lysine were greater (all *p* < 0.05). In postprandial phase, aspartic acid, threonine, valine and lysine concentrations were greater at 2 h compared to 0.5 h, whereas tyrosine levels were greater at 0.5 h compared to 2 h (all *p* < 0.05).

Interestingly, we found no change in MPS in response to ingestion of a bolus of whey protein in either of the genotypes from 0–0.5 h or from 1.5–2 h after feeding (Fig. 2B). Moreover, for WT vs. Apoe KO mice *p* = 0.105, thus, there was no significant difference in puromycin incorporation in the postprandial period (Fig. 2B).

**Figure 6:**
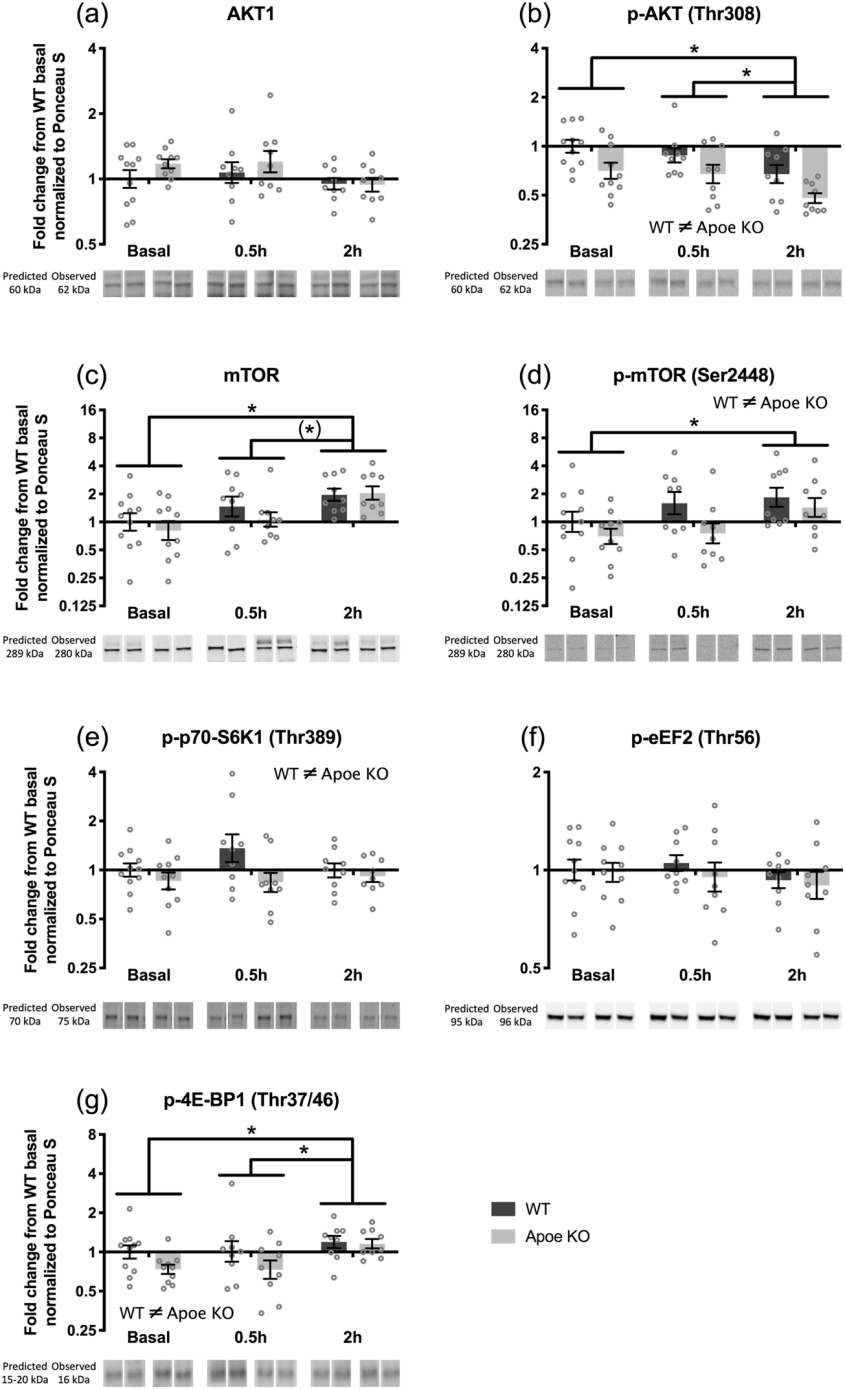
Hypertrophy signaling pathway during the acute trial. Protein expression of AKT1 (**A**), p-AKT(Thr308) (**B**), mTOR (**C**), p-mTOR(Ser2448) (**D**), p-p70-S6K1(Thr389) (**E**), p-eEF2(Thr56) (**F**), and p-4E-BP1(Thr37/46) (**G**) in WT (dark bars) and Apoe KO (grey bars) mice at basal (WT n = 12, Apoe KO n = 10), 0.5 h (WT n = 9, Apoe KO n = 9) and 2 h (WT n = 9, Apoe KO n = 9) after whey protein ingestion. Data were normalized to Ponceau S and expressed as fold change from WT basal and individual values are shown with bars indicating geometric means ± back-transformed SEM. (*) trend for a difference between time points 0.05 ≤ *p* < 0.10, * difference between time points, *p* < 0.05. Samples were loaded in a randomized order, repeated twice on the same gel. Therefore, representative images of the bands were cropped and reorganized to represent the respective time point and mouse. Expected and observed band size are indicated for each target.

For the hypertrophy signaling in response to whey protein feeding, we found no changes in total AKT1 expression (Fig. 6A), but a significant decrease in p-AKT(Thr308) was observed at 2 h compared to basal (*p* = 0.002) and 0.5 h (*p* = 0.017) (Fig. 6B). Furthermore, p-AKT(Thr308) expression was in general lower in the Apoe KO compared to WT mice (*p* < 0.001). Total mTOR increased at 2 h compared to basal (*p* < 0.001) and with a trend compared to 0.5 h (*p* = 0.056) in both WT and Apoe KO mice after whey protein ingestion (Fig. 6C). p-mTOR(Ser2448) was increased at 2 h compared to basal (*p* = 0.023) (Fig. 6D). In general, Apoe KO mice had lower levels of p-mTOR(Ser2448) compared to WT mice (*p* = 0.025). For p-p70-S6K1(Thr389) an overall greater expression was seen in WT compared to Apoe KO mice (*p* = 0.023) with no specific effect of time (Fig. 6E). The expression of p-eEF2(Thr56) did not change (Fig. 6F), when stimulating with whey protein. p-4E-BP1(Thr37/46) increased after 2 h compared to basal (*p* = 0.045) and 0.5 h (*p* = 0.039) and the p-4E-BP1(Thr37/46) expression was significantly higher in the WT mice compared to Apoe KO mice (*p* = 0.039) (Fig. 6G).

**Figure 7:**
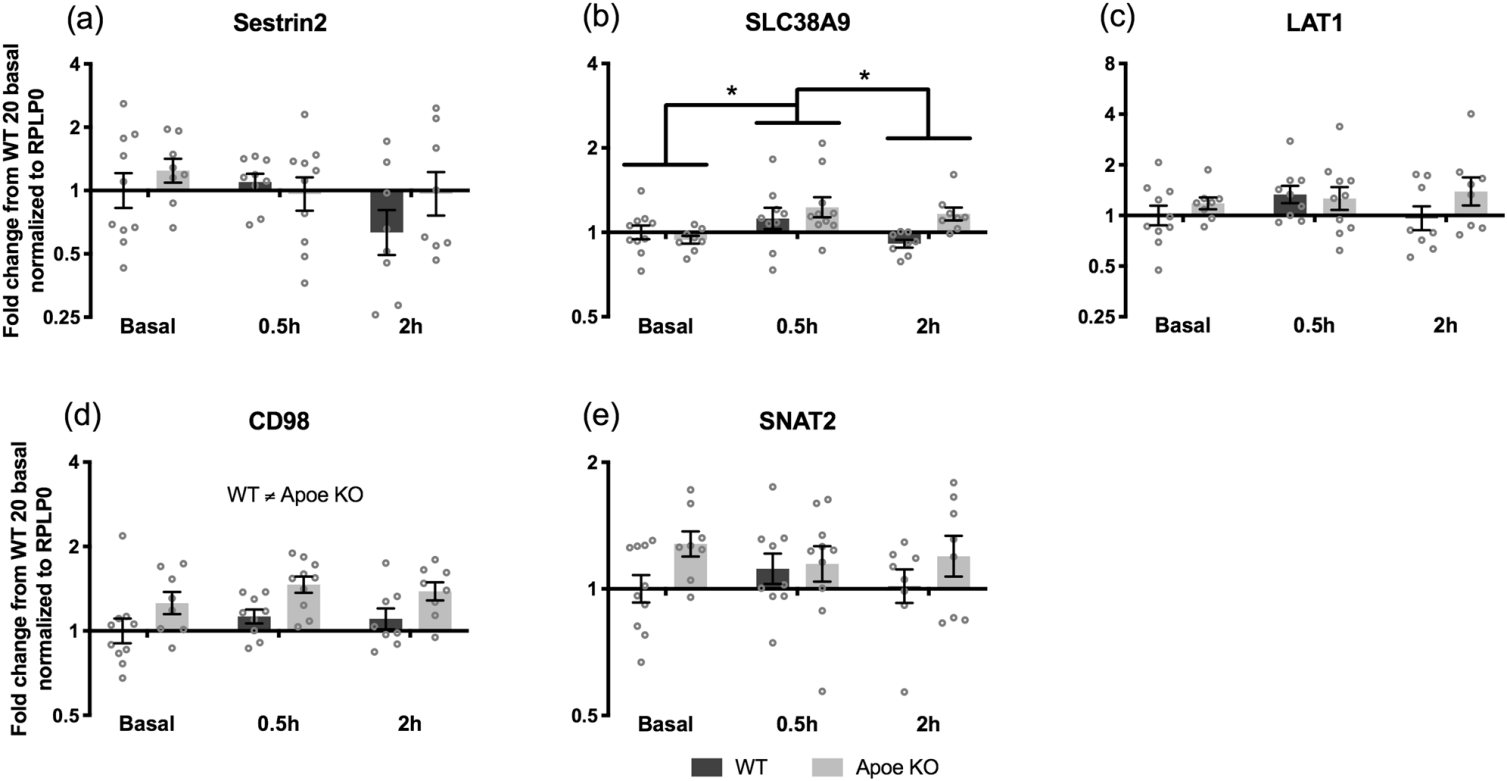
Amino acid transporters and sensors during the acute trial. mRNA expression of Sestrin2 (**A**), SLC38A9 (**B**), LAT1 (**C**), CD98 (**D**), and SNAT2 (**E**) in WT (dark bars) and Apoe KO (grey bars) mice at basal (WT n = 10, Apoe KO n = 8), 0.5 h (WT n = 9, Apoe KO n = 9) and 2 h (WT n = 8, Apoe KO n = 8) after whey protein feeding. Data were normalized to RPLP0 and expressed as fold change from WT basal and individual values are shown with bars indicating geometric means ± back-transformed SEM. * difference between time points, *p* < 0.05.

For the amino acid transporters and sensors no change in Sestrin2 mRNA expression was found after whey protein feeding (Fig. 7A). Whey protein feeding increased overall SLC38A9 mRNA expression at 0.5 h compared to basal (*p* = 0.012) and a trend was found for higher expression at 0.5 h compared to 2 h (*p* = 0.097). Furthermore, there was a trend for an effect of genotype (*p* = 0.090) and interaction (genotype x time) (*p* = 0.078) (Fig. 7B). The mRNA expression of the amino acid transporter LAT1 did not change in response to whey protein feeding (Fig. 7C), but the mRNA expression of the associated protein CD98 was in general significantly higher in Apoe KO compared to WT control mice (*p* < 0.001) (Fig. 7D). A trend towards a genotype effect *p* = 0.052 was seen for SNAT2 mRNA expression (Fig. 7E).

During the acute trial at 20 weeks of age mRNA expressions of targets for amino acid deprivation GCN2 and ATF4 were in general higher in the Apoe KO mice compared to WT mice (GCN2 *p* < 0.001, ATF4 *p* = 0.003) (Fig. 5C+D). There were no changes in GCN2 or ATF4 mRNA expression in response to whey protein ingestion.

### Gut microbiome

**Figure 8:**
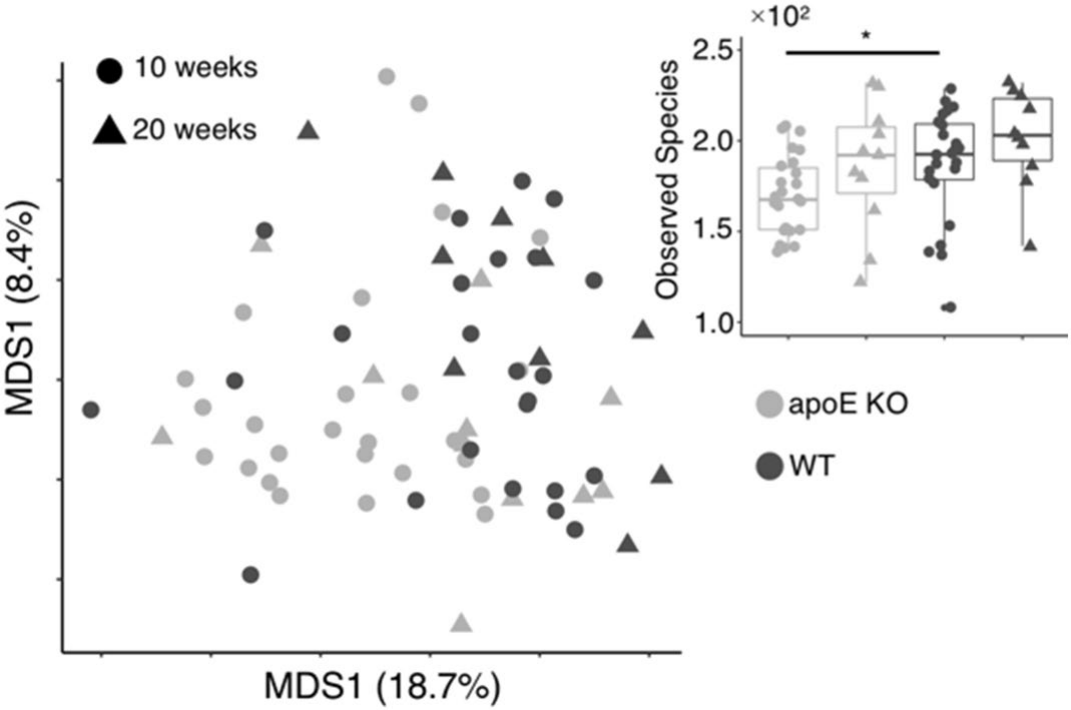
Multidimensional scaling plot of GM profiles (**A**) based on Sørensen-Dice dissimilarities and alpha-diversity summary based on observed species index (**B**). Analyses were based on 90,000 reads per sample on zOTUs aggregated to the species level. * difference in observed species *p* < 0.05. Week 10; WT n = 10, Apoe KO n = 11 and week 20; WT n = 24, Apoe KO n = 26.

GM compositions potentially implicated in the expression of the latter mRNAs were profiled using high-throughput sequencing of 16S rRNA partial-gene. Beta-diversity (Sørensen-Dice distance) revealed significant compositional changes between mice strains (Adonis test *p* = 0.001). This accounted for 6.9% of the GM variance and was associated to variations in bacterial members that composed 18–41% of the average relative distribution (Fig. 8A, details in Supplemental Table S1). Similarly, the experimental design (weeks 10 to week 20) captured up to 3.6% of the GM variance (Adonis test *p* = 0.01), and being associated to fluctuations in bacterial members that composed 71–88% of the average relative distribution (Fig. 8A, details in Supplemental Table S1). In relation to Alpha-diversity, the number of observed species differed significantly (t-test *p* = 0.03) only between ApoE KO and WT at 10 weeks.

## Discussion

In the current study, the effect of an unhealthy lipoprotein distribution was investigated in the basal state and on the anabolic response to whey protein feeding by comparing WT and Apoe KO mice. Mixed MPS was assessed through puromycin incorporation and targets of the mTORC1-cascade explored hypertrophy signaling. Furthermore, gene expression of key amino acid sensors, and targets implicated in the amino acid deprivation response pathway was measured. No differences in basal MPS between the Apoe KO and WT mice were seen, and surprisingly no increases in MPS in response to whey protein feeding could be observed. Despite this, both mTOR and 4E-BP1 phosphorylation were increased 2 h after whey protein feeding indicating an anabolic response. According to our hypothesis, a general lower expression of AKT1, mTOR, p70-S6K1 and 4E-BP1 phosphorylation in the Apoe KO mice were seen. Furthermore, the Apoe KO mice had increased mRNA levels of GCN2 and ATF4, two targets implicated in the amino acid deprivation response, compared to the WT mice indicating amino acid deprivation in the Apoe KO mice.

The purpose of this study was to investigate if the unhealthy LPD in the Apoe KO mice would affect the anabolic response to a bolus of whey protein. To our knowledge, nobody has investigated the effect of an altered plasma lipoprotein profile, on MPS in mice or humans, making it difficult to compare the current results with previous studies. An unhealthy LPD is often related to elevated triglycerides in plasma. Few studies have tested the effect of acutely elevated free fatty acid in plasma with mixed results^28,29^. We observed no differences between the genotypes on puromycin incorporation but a trend for increased puromycin incorporation and thereby increased MPS from 10 to 20 weeks in the basal state in WT and Apoe KO mice (Fig. 2A). Possibly, the altered MPS could be an effect of growth.

Dijk and colleagues have also applied puromycin labeling to study MPS in response to protein feeding over a period of 30 min in mice^15,35^. The types and amounts of protein differed between the current study and their studies. In the study by Dijk et al. from 2017, the mice were fed a protein solution also containing additional leucine, fat and carbohydrates, while the amount of protein corresponded to what was given in this study (3.8 mg whey protein × g LBM^−1^). A rise in puromycin incorporation was found at 45–75 min after ingestion^35^. Later in 2018, Dijk and colleagues investigated mice that were fed leucine alone or whey plus leucine. Leucine alone failed to induce an increase in protein synthesis, but leucine enriched whey protein increased protein synthesis at 0.5–1 h after ingestion^15^. The amount of total protein and leucine in Dijk et al. 2018 was greater than what we provided, and the total amounts of calories were greater. A mixed macronutrient solution in van Dijk et al. 2017 could possibly induce a greater effect than what we saw in response to whey protein ingestion alone (Fig. 2B)^35^. Furthermore, a rise in puromycin incorporation could have occurred had we chosen a time interval between 0.5 to 1.5 h, since just a 30-min shift has shown to reveal a different MPS in the post-prandial response to whey protein^15^. The early (0–0.5 h) and later (1.5–2 h) time points were chosen to cover the immediate post-prandial period, as a lacking MPS response to protein feeding has been found later in the post-prandial phase^36^.

We had hypothesized that the MPS was higher in the WT compared to the Apoe KO mice in response to protein feeding. The amount of whey protein ingested by the mice in this study corresponded to approximately 20 g for humans, and this has been shown to be sufficient to increase MPS in healthy young and old men^37–39^. An increase in intramuscular EAA was seen over the postprandial phase after whey administration, whereby a prior increased extracellular plasma EAA concentration known to be important for MPS^37,40^ must have preceded. The lack of anabolic response to whey protein in this study could be due to a different metabolism in the mice compared to humans, e.g. that a comparable amount of protein was not enough to elicit an increase in MPS in mice.

Alterations in circulating blood cholesterols have previously been linked to gut microbiome alterations^31^. Similarly, ApoE KO mice have been found to harbor a different gut microbiome profile than WT mice, which was accompanied by significant differences in HDL cholesterol as well^41^. Surprisingly, in the present study, no significant gut microbiome differences were observed between Apoe KO and WT (Fig. 8).

The anabolic signaling involved in the MPS response was evaluated by measuring changes in phosphorylation of select targets from the mTORC1-signaling cascade to elucidate changed signaling between the WT and Apoe KO mice that might not be revealed by differences in MPS. From 10 to 20 weeks of age total AKT1 did not change, but we found a decrease in total mTOR (Fig. 3), which could be due to decreased growth rate at 20 weeks of age. There was no difference in these proteins between the WT and Apoe KO mice.

In the acute study at 20 weeks of age, we found lower expression of p-mTOR (Ser2448), p-4E-BP1 (Thr37/46) and p-p70-S6K1 (Thr389) in the Apoe KO mice compared to the WT mice (Fig. 6). This indicates a general lower hypertrophy signaling in the Apoe KO mice, which however was not seen on the MPS results at the selected time points. We found a decrease in p-AKT (Thr308) at 2 h compared to basal (Fig. 6), indicating no contribution of the insulin signaling pathway on our mTORC1-signaling cascade. Furthermore, we saw an increase in both total mTOR, p-mTOR (Ser2448) and p-4E-BP1 (Thr37/46) in response to whey protein feeding at 2 h in line with observations from others^3,15,42^. Contrary to this, we did not see any changes in eEF2 (Fig. 6), where a decrease in the phosphorylation status in response to whey protein feeding was expected^42^, as the phosphorylated eEF2 is inhibiting protein synthesis^43^. The mTORC1 signaling pathway data indicate that protein synthesis was stimulated by the whey protein ingestion even though it was not seen on the MPS from the puromycin incorporation data.

Looking at the intracellular amino acid sensors from 10 to 20 weeks of age, we found overall decreases in Sestrin2, CD98 and SNAT2 mRNA expression (Fig. 4), which indicate a potential for an altered protein expression of these targets. This might again be due to decreased growth rate at 20 weeks of age and therefore a decreased demand for amino acids. Some studies have reported increases in both LAT1 and CD98 mRNA expression in response to feeding in humans^44^, whereas other studies did not detect an increase in LAT1 and CD98 mRNA expression in response to ingestion of a whey protein bolus^45,46^. A feeding response on LAT1 and CD98 mRNA expression was not seen. Instead a response to whey protein feeding was found for SLC38A9 mRNA at 0.5 h compared to basal (Fig. 7B), which is in line with Graber et al. 2017, where SLC38A9 mRNA expression also increased significantly at 1 h post EAA in humans ingesting 10 g of EAAs^45^. In the current study, significantly higher mRNA levels of CD98 and SNAT2 mRNA expression in the Apoe KO mice compared to the WT mice were seen (Fig. 4). This might be a way to overcome the general lower signaling level of the mTORC1 signaling pathway in the Apoe KO mice, by improving the intracellular amino acid transport and sensing capacity, anticipating that the mRNAs translate into functional amino acid transporters. Interestingly, we did find higher intramuscular glutamine levels in the Apoe KO vs. WT mice (Table 1). This could indicate a greater activity of the SNAT2 transporter that ensures inward transport of glutamine, which is needed for the exchange transport of especially BCAA through the LAT1/CD98 complex^16^.

Two targets involved in the amino acid deprivation response, GCN2 and ATF4, were upregulated in the Apoe KO mice (Fig. 5). When amino acids are limited in the cell, GCN2 becomes activated by binding uncharged transfer-RNAs leading to ATF4 transcription^20^. The upregulation could indicate that the Apoe KO mice in general were amino acid deprived compared to the WT mice and was further supported by the higher CD98 and SNAT2 mRNA expressions discussed above. This could also be a response to the elevated ATF4 mRNA expressions as ATF4 functions as transcription factor for at least SNAT2^22^. Despite the amino acid deprivation response was activated to a higher degree in the Apoe KO mice, we did not see distinct intramuscular AA concentrations (Table 1). Thus, potentially the altered GCN2 and ATF4 response could ensure proper AA concentrations, which would also explain why the LBM was not distinct between the Apoe KO and WT mice.

GCN2 mRNA expression decreased from 10 to 20 weeks in both WT and Apoe KO mice (Fig. 5A). The same pattern was seen in ATF4 mRNA, where the mRNA expression also decreased from 10 to 20 weeks, with the general Apoe KO AFT4 mRNA expression being higher than WT (Fig. 5B). The decrease from 10 to 20 weeks of age could be due to decreased growth rate at 20 weeks of age, which was also reflected in a lower total level of mTOR at 20 compared to 10 weeks, and therefore a decreased demand for amino acids^47^. There is previous evidence showing that GCN2-ATF4 signaling pathway may also induce responses against pathogenic bacteria^48^ as well as inhibiting inflammasome activation in the gut^49^. In our experimental setup, we were unable to link the increased levels of expression of GCN2 and ATF4 mRNA expression with the abundance profiles of GM members and this task remains to be elucidated in future studies. Again, we would expect the GCN2 and ATF4 mRNA expressions to be higher after the overnight fast and decrease in response to whey protein feeding^44^. Not many groups have evaluated the amino acid deprivation response to re-feeding, why we lack data to compare our study to.

The distinct signaling pattern of the Apoe KO and WT mice are in contrast to the phenotypes. Body weight and LBM increased from 10 to 20 weeks of age but was not different between Apoe KO and WT mice. It must be noted that a mouse at 20 weeks of age is still a rather young mouse. Therefore, the growth of a young mouse could be ensured despite being affected by processes interfering with the anabolic- and amino acid sensing mechanisms. It should be noted that the scope of the present study was to study these anabolic processes and amino acid sensing to protein feeding, but still acknowledge that muscle mass is also controlled as well by catabolic processes involved in protein degradation. Future studies could elucidate if catabolic processes are enhanced having an unhealthy LPD. In the present study, the lower level of mTORC1 signaling in the Apoe KO could potentially facilitate a greater degree of autophagy. Yet, we still have identical muscle phenotypes of the Apoe KO and WT mice.

Although we are studying mechanism which in humans could contribute to age related loss of muscle mass, the Apoe KO mouse in the current study is not a model of aging. It would have been beneficial to study the Apoe KO mouse at an adult or aged stage. However, atherosclerotic development of the Apoe KO at an adult stage is too severe, whereby a great number of animals would have to be euthanized due to humane endpoints. Therefore, we can only speculate that the lower anabolic signaling and amino acid deprivation response we clearly see in the Apoe KO mice would be reflected in LBM phenotype, if it had been possible to study the mice at an older age.

## Conclusion

The unhealthy LPD in the Apoe KO mice did neither affect basal MPS nor reveal a different post-prandial MPS after protein feeding compared to WT mice at the selected time points. Also, the gut microbiome did not differ between Apoe KO and WT. Although, the primary results from this study did not clarify our hypothesis on the MPS data, we did observe lower levels of important phosphorylation targets implicated in the protein synthesis pathway in the Apoe KO mice with unhealthy LPD. Likewise, GCN2 and ATF4 mRNA expressions were higher in the Apoe KO mice indicating that the mice were metabolically challenged and amino acid deprived. Furthermore, CD98 mRNA expression were elevated in the Apoe KO mice at all time points, giving a mechanism to compensate for the amino acid deprivation. Thus, altogether the Apoe KO mice with an unhealthy LPD seemed to be anabolic challenged and had an increased activation of the amino acid deprivation response pathway.

## Materials and methods

### Animals

Male Apoe knockout (Apoe KO) mice B6.129P2-*Apoe*^*tm1Unc*^ N11 were obtained from Taconic (Borup, Denmark). The mouse model was originally developed through embryonic transfer^51^ and the line is maintained by inbreeding homozygous mice. As control mice, male wild type (WT) C57BL/6JBomTac mice were used with same genetic background (C57BL/6) as the Apoe KO mice. The animals (WT N = 48, Apoe KO N = 48) were purchased at 10 weeks of age, group housed, and kept on a 12-h light/dark cycle at constant room temperature. Both WT and Apoe KO mice were feed ad libitum water and standard rodent chow (#131003, Altromin, Lage, Germany).

### Experimental trial

All animal experiments were conducted in accordance with Danish national guidelines, approved by the Danish Animal Experiments Inspectorate, Ministry of Justice under trial number 2015-15-0201-00805 and followed the ARRIVE guidelines.

On the experimental days, four experiments were performed in parallel on either WT or Apoe KO mice. The experimental days were alternating between either WT mice or Apoe KO mice. The mice were randomly divided into four groups that were euthanized at; 10 weeks of age (10 weeks), 20 weeks of age (20 weeks) in post-absorptive state, and at 20 weeks of age in post-prandial state 0.5 h (0–0.5 h) and 2 h (1.5–2 h) after whey protein feeding (Supplemental Figure S2). Twelve mice of each genotype for each time point were bought. However, due to humane endpoints some mice died or were euthanized before analysis. By this we ended up having; 10 weeks fasted n = 12 for both Apoe KO and WT, 20 weeks fasted n = 10 for Apoe KO and n = 11 for WT, 0–0.5 h n = 10 for Apoe KO and n = 9 for WT, and 1.5–2 h n = 9 for both Apoe KO and WT. To avoid influence of circadian fluctuations, experiments for all mice were performed at the same time of day with measurements of basal rates being commenced between 10.00 and 10.15 and with time point 0 h being between 11.00 and 11.15.

On experimental days, the mice were weighted and had an MRI scan (EchoMRI4in1-500, EchoMRI) to determine fat and lean body mass (LBM). Thereafter, the mice were intraperitoneally anaesthetized with ketamine-xylazine 20 μl × g bodyweight^−1^ (Ketamin 25 mg × ml^−1^, xylazin 10 mg × ml^−1^). Muscle protein synthesis response to whey feeding was measured by the use of puromycin labeling. When the mice were fully anaesthetized, 2.2 μl × g bodyweight^−1^ puromycin (#P-1033-SOL 10 mg/ml, AG Scientific, CA, USA) was i.p. injected. 30 min after, the mice were euthanized by cervical dislocation.

In the whey groups, 0–0.5 h and 1.5–2 h, a whey bolus of 3.8 mg × g LBM^−1^ (Lacprodan DI-9224, Arla Foods Ingredients, Viby J, DK) was provided by oral gavage. The dose corresponded to approx. 20 g in humans^52^. Anesthesia was performed immediately hereafter (0–0.5 h group) or at 1.5 h after whey protein feeding (1.5–2 h group). The mice were euthanized 30 min after puromycin injection.

Blood was sampled by cardiac puncture, mixed with 3.2% sodiumcitrate 9:1 v/v and kept on ice for at least 10 min to prevent clotting before the blood samples were spun down for 10 min at 2000 g, hereafter the plasma was collected. Gastrocnemius and tibialis anterior muscles from the left hind leg were dissected out. Gastrocnemius was snap frozen in liquid nitrogen. All samples were stored at − 80 °C until further analysis.

### Western blotting

Approximately 20 mg gastrocnemius muscle was placed in a 2 ml microvial with a screw cap (BioSpec) containing one silicium-carbide crystal, five 2.3 mm steel beads and 30 μl homogenization buffer per mg muscle tissue. The homogenization buffer at pH 7.5 consisted of 50 mM Tris-Base, 1 mM EDTA, 1 mM EGTA, 10 mM beta-glycerophosphate, 50 mM NaF, 300 mM sucrose, and immediately before use 1 protease inhibitor tablet (Complete, Roche), 1 phosphatase inhibitor tablet (PhosSTOP Complete, Roche, Switzerland), 10 μl β-mercaptoethanol and 100 μl 10% Triton-X-100 was added per 10 ml buffer. The muscle sample was homogenized using the FastPrep 24 shaker (MP Biomedicals, Illkirch, France). Samples were spun 3 min at 2000 g at 4 °C, and the supernatant collected for analysis. Protein concentration of the supernatant was determined by Bradford protein assay. The supernatant was diluted 1:1 in loading buffer (1.25 M Tris, pH 6.8, 25% (v/v) glycerol, 2.5% SDS, 2.5% (v/v) β-mercaptoethanol, and 0.2% (w/v) bromophenol blue). Samples for loading were heated at 95 °C for 5 min before 11 μg protein per sample was loaded on SDS–polyacrylamide gels. Samples were loaded in duplicates, and a standard sample was loaded in triplicates on each gel to allow comparison between the gels. Samples for puromycin analysis were run on 10.5–14% gels, whereas samples for signaling pathway analysis were run on 7.5% and 12% gels (Criterion TGX Stain-Free Protein Gel, Bio-Rad, CA, USA). SDS- PAGE was run for 1 h 10 min at 150 V in electrophoresis buffer (25 mM Tris-base, 190 mM glycine, and 3.5 mM SDS). Gels with samples for signaling pathways were cut horizontally making it possible to put three gels on the same PVDF membrane and thereby minimizing the differences in probing for a specific target on multiple membranes. The separated proteins were transferred to low fluorescent PVDF membranes (Immun-Blot LF PVDF Membrane, Bio-Rad, CA, USA) by wet transfer for 1 h at 50 V in transfer buffer (10 mM CAPS, pH 11.0, and 10% (v/v) methanol). The membranes were blocked in 20% (v/v) Odyssey PBS buffer and then incubated over night at 4 °C with primary antibodies against puromycin (Eq0001, Kerafast, MA, USA), mTOR (#2983, Cell Signaling, MA, USA), p-mTOR(ser2448) (#2971, Cell Signaling, MA, USA), AKT1 (#3967, Cell Signaling, MA, USA), p-AKT(Thr308) (#2965, Cell Signaling, MA, USA), p-p70-S6K1(Thr389) (#9206, Cell Signaling, MA, USA), p-eEF2 (#2331S, Cell Signaling, MA, USA), p-4E-BP1(Thr37/46) (#2855, Cell Signaling, MA, USA) in 10% Odyssey PBS buffer. All primary antibodies were diluted 1:1000 except for p-eEF2 (1:2000). The following day, the membranes were washed 3 × 5 min in TBST before being incubated for 1 h with secondary antibodies Goat anti mouse IgG (Alexa 680, A21057, Invitrogen, MA, USA) and goat anti rabbit IgG (Dylight 800, 35571, Thermo Scientific, MA, USA) diluted 1:10.000 in 10% Odyssey PBS buffer. Images of the membranes were obtained on an Odyssey scanner (Odyssey Infrared Imaging System; Li-Cor Bio-sciences, Lincoln, NE, USA) and the expression of protein bands were quantified by lane plots on ImageJ, with the assessor blinded towards group and time point allocation. For puromycin incorporation the AUC of a full lane plot were obtained. Finally, Ponceau S staining of all membranes was performed for normalization of the protein expression. On all membranes a standard sample was loaded three times/in triplicates (see Supplemental Figure S3). The intensity from these triplicates of the standard sample on each membrane was used to normalize the intensity of Ponceau S as well as the specific protein target across membranes. Thereby any differences in Ponceau S staining efficiency or protein blotting efficiency between membranes were accounted for. Representative image of blots and Ponceau S staining is shown in Supplemental Figure S3.

### Muscle free amino acid concentration

On a randomly chosen subset of muscle tissue samples at 20 weeks of age (n = 4 at each time point; basal, 0.5 h and 2 h, respectively) of each mouse type the intramuscular muscle free amino acid concentration (MFAA) was measured. Liquid chromatography tandem mass-spectrometry (LC–MS/MS) (TSQ Quantiva; Thermo Fisher Scientific, San Jose, CA) was used to measure the amino acid concentrations as described elsewhere^53^. For the analysis, approximately 20 mg of muscle tissue per sample was used. Importantly, each of the individual 19 amino acids was quantified by adding a standard of their own stable isotopically labeled internal standard (uniformly labeled-^13^C/^15^N) to the samples.

### RT-qPCR

RNA was purified from approximately 10 mg gastrocnemius muscle. The muscle samples were homogenized in 1000 μl TRIreagent in a 2 ml microvial with a screw cap (BioSpec) containing one silicium-carbide crystal and five 2.3 mm steel beads (Molecular Research Center, Cincinnati, OH, USA) using the FastPrep 24 shaker (MP Biomedicals, Illkirch, France). 1-bromo-3-chloropropane (BCP) was added to each tube, and the tubes were centrifuged to separate the water phase containing the RNA. Isopropanol was added to precipitated RNA from the aqueous phase, tubes were spun, and the remaining pellet washed in 75% ethanol and dissolved in RNAse free water; precipitation, wash and dissolving was performed two times. The RNA concentration and purity was measured by UV spectrophotometry. 500 ng RNA was converted to cDNA (20 μl) using the Omniscript Reverse Transcriptase Kit. The cDNA samples were diluted 20 times in TE buffer containing 1 ng × μl^−1^ salmon DNA. PCR plates were loaded (25 μl final volume) with 5 μl diluted cDNA and a standard curve and PCR mastermix (Quantitect SYBR Green PCR from Qiagen, Hilden, Germany) and 100 nM PCR primers for Ribosomal protein large P0 (RPLP0), glyceraldehyde-3-phosphate dehydrogenase (GAPDH), SNAT2, LAT1, CD98, SLC38A9, Sestrin2, ATF4 and GCN2 (Table 2). The PCR program consisted of denaturation at 95 °C for 10 min and 50 cycles of melting at 95 °C for 15 s, annealing at 58 °C for 30 s, elongation at 63 °C for 90 s. After the last amplification cycle, specificity of the PCR product was confirmed by a melting curve analysis (95 °C for 60 s, 55 °C for 30 s, and slow heating to 95 °C). Ct values were related to the standard curve consisting of known amounts of oligonucleotides identical to the PCR products. Data was normalized to RPLP0. As a control, Glyceraldehyde 3-phosphate dehydrogenase (GAPDH), another often constitutively expressed mRNA, was normalized to RPLP0 (Supplemental Figure S4). GAPDH normalized to RPLP0 showed no difference between any groups or changes over time, why we accept the normalization targets.

**Table 2:** Sequences of PCR primers for RPLP0, GAPDH, SNAT2, LAT1, CD98, SLC38A9, Sestrin2, ATF4, and GCN2 mRNAs.

Target	Ref. sequence	Sense primer	Antisense primer
RPLP0	NM_053275.3	GGAAACTCTGCATTCTCGCTTCCT	CCAGGACTCGTTTCTACCCGTTG
GAPDH	NM_002046.4	CCTCCTGCACCACCAACTGCTT	GAGGGGCCATCCACAGTCTTCT
SNAT2	NM_175121.3	GGAGACGCTGCCGTGAGGTG	GCGGGCTTCCTTTTGTCCTTG
LAT1	NM_011404.3	GCGGGCTGCCTGTCTACTTCTTT	CTCCTGAGGTACCACCTGCATCAAC
CD98	NM_001161413.1	TTTAGCTACGGGGATGAGCTTGG	TTGAGGCTTACAGGTCTTGGGATG
SLC38A9	NM_178746.4	CATTCCCTTCGCCTCCATTACC	GGATGTCACTGCTGGGGAAGTTGT
Sestrin2	NM_144907.1	AGCGGGGACCCACTGAACAACTC	CTCCTGCGAAGCCCCCTCATC
ATF4	NM_009716.3	GCAACCCCCACCGGCCTAA	TGTTGTGGGGCTTTGCTGGATT
GCN2	NM_013719.3	CAAGCTCAGCCAAGTCTACGTCATTC	GCTTCCACTTCTCTCCTAGTCAGCTTC

### Cholesterol analysis

HDL- and LDL cholesterol were measured to confirm the phenotype of the Apoe KO mice using the HDL and LDL/VLDL Cholesterol Assay Kit (ab65390, Abcam, UK). The plasma samples were divided into HDL- and LDL/VLDL cholesterol fractions by mixing 25 μl sample with 75 μl dH_2_O, adding 100 μl 2X Precipitation Buffer. After 10 min incubation at room temperature, the samples were centrifuged at 2000 g for 10 min at 20 °C. All supernatant, the HDL fraction, was transferred to a separate tube and the remaining pellet was suspended in 200 μl PBS, giving the LDL/VLDL fraction. All samples were loaded in duplicates on a 96 well plate. To ensure an absorbance reading within the standard curve, the HDL cholesterol samples were loaded in 10 μl and 15 μl of WT and Apoe KO, respectively. Likewise, of the LDL/VLDL cholesterol samples 30 μl and 10 μl were loaded from the WT and Apoe KO, respectively. Assay buffer was added to each well giving a volume of 50 µl in total. 50 µl Total Cholesterol Mix was then added to each well and the plate was mixed and incubated at 37 °C for 60 min. Thereafter, the plate was measured at OD570 nm and cholesterol concentration calculated. Total cholesterol was calculated as the sum of HDL- and LDL/VLDL cholesterol concentrations.

### Gut microbiome

At least two pellets of colon fecal samples were collected directly from the mouse on the day prior to the experimental trial. Fecal samples were immediately frozen and stored at -80˚C until further analysis. Total DNA was extracted and the gut microbiome composition was determined by 16S rRNA gene amplicon sequencing targeting the 16S rRNA gene V3-region by Illumina NextSeq high throughput sequencing as previously described^32^.

The raw dataset containing pair-ended reads with corresponding quality scores were merged and trimmed using settings previously described^32^. Finding unique reads and deconvoluting from chimeric reads and constructing *de-novo* zero-radius Operational Taxonomic Units (zOTU) was conducted using the UNOISE pipeline^54^ coupled to the EZtaxon 16S rRNA gene collection as a reference database^55^. Downstream analyses (see Statistical section) were based on a contingency table rarefied to 90,000 random sequences per sample summarized to species level.

### Statistical analysis

All data was log transformed and checked for normality by the Shapiro–Wilk test and for equal variance by the Brown-Forsythe test. MRI and MFAA data were not log transformed to obtain a normal distribution. Except for gut microbiome, data was analyzed using two-way ANOVA without repeated measurements. Whenever significant effects of time, group/mice or interaction were seen, Student–Newman–Keuls post-hoc test was applied (SigmaPlot, ver. 13.0, Systat Software Inc, CA, USA). For gut microbiota analysis the influence of mice strain and time on alpha-diversity was assessed with two-tailed *student’s t-*test, while beta-diversity was analyzed using Adonis test based on Sørensen-Dice dissimilarities as implemented in the *Vegan* R-package^56^. Differences in the ratio of species frequencies between experimental groups (up to 362 evaluated species for each group) were analyzed using G-test (Goodness of fit) and adjusting all P-values for multiple testing with Bonferroni correction.

*P*-values below 0.05 were considered significant and trends are reported for *p*-values between 0.05 and 0.10. Data are shown as geometric mean ± back-transformed standard error of mean (SEM) unless otherwise specified.

## Supplementary Information

Supplementary Information.
