# Supplementary material for: Impaired skeletal muscle hypertrophy signaling and amino acid deprivation response in Apoe knockout mice with an unhealthy lipoprotein distribution
Source: Sci Rep. 2021 Aug 12;11:16423. doi: 10.1038/s41598-021-96000-8 (PMC8360952; doi:10.1038/s41598-021-96000-8)
Supplement: Supplementary file 1 — Supplementary Information. [file 41598_2021_96000_MOESM1_ESM.pdf]

SUPPLEMENTAL MATERIAL

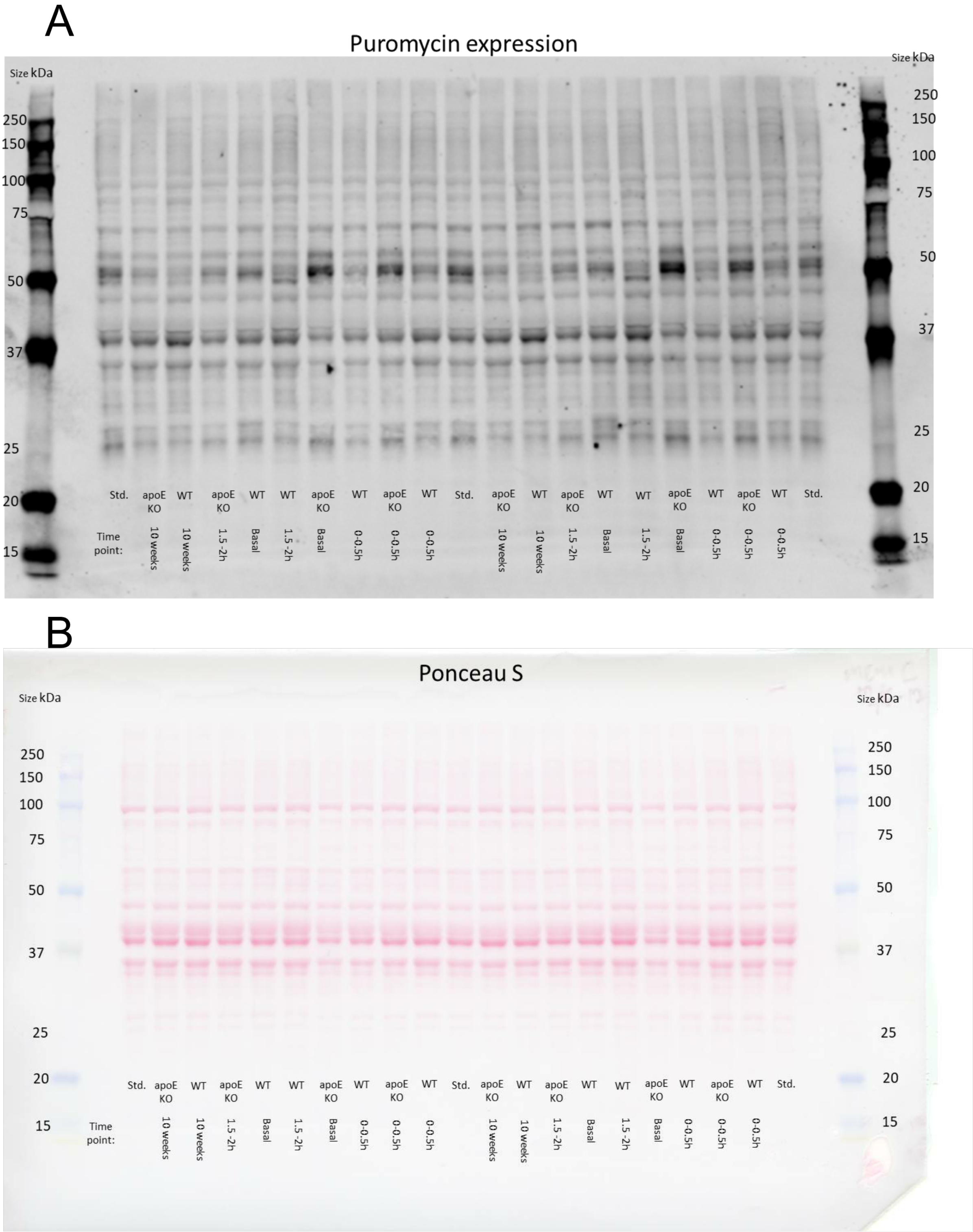

Supplemental figure S1: Representative blots of puromycin incorporation (A) and ponceau S staining (B). Samples were loaded in a randomized order, repeated twice on the same gel. Genotype and time points are indicated on the figure.

SUPPLEMENTAL MATERIAL

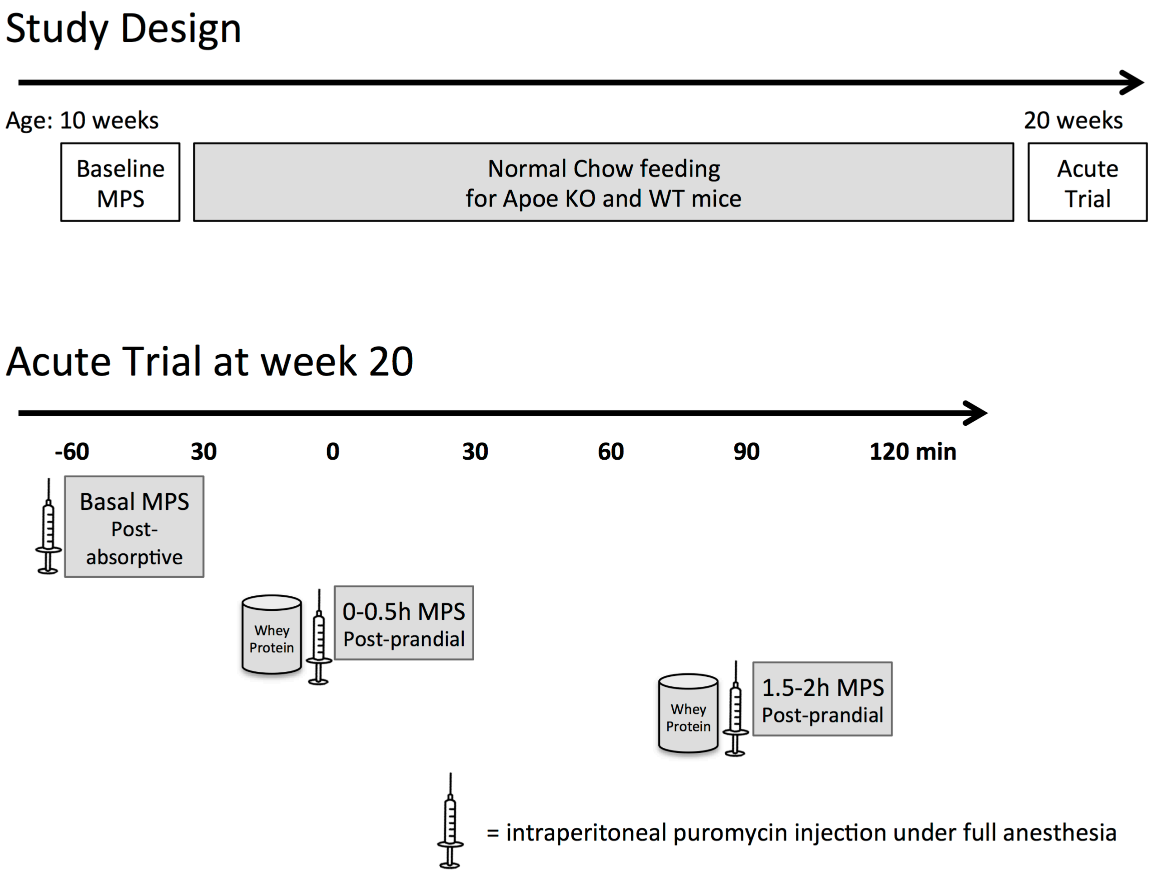

Supplemental figure S2: Study design overview.

SUPPLEMENTAL MATERIAL

AKT1 – representative blots from figure 3a

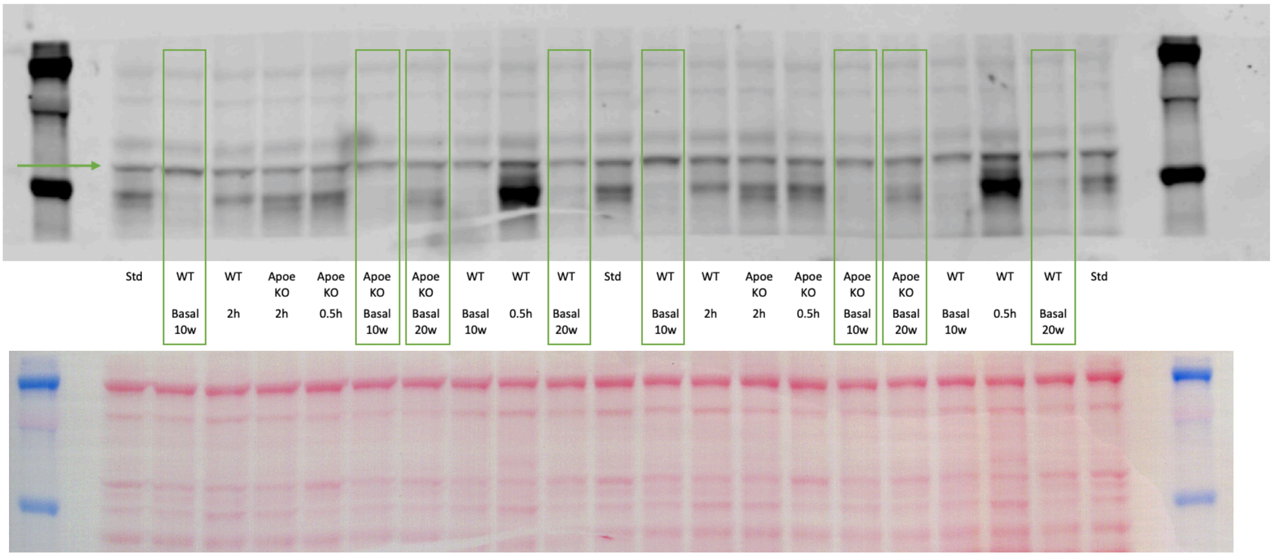

mTOR – representative blots from figure 3b

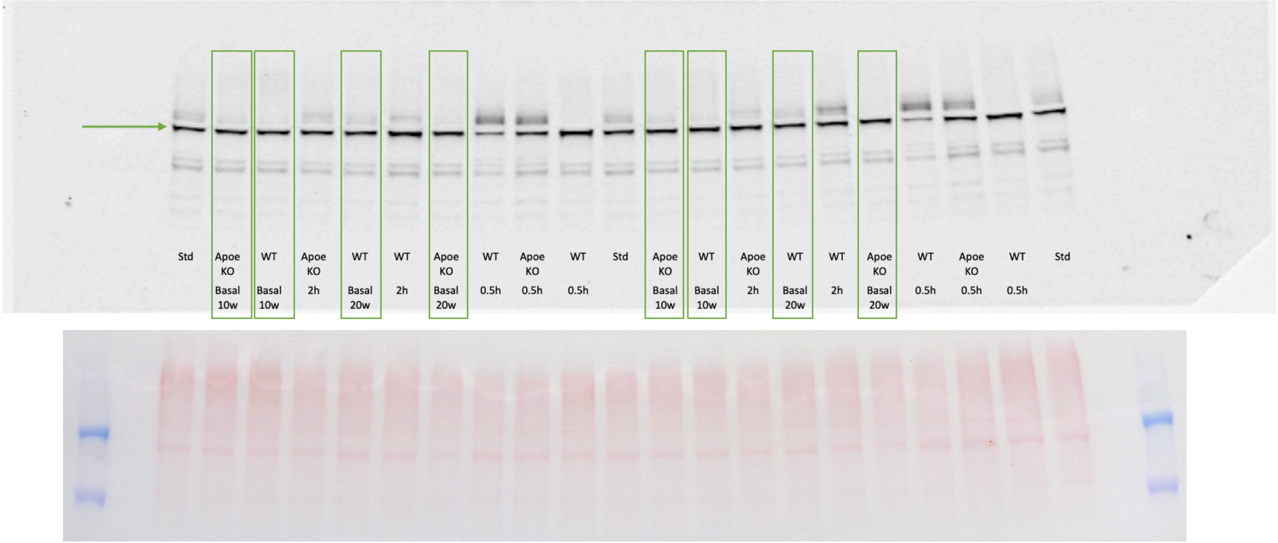

AKT1 – representative blots from figure 6a

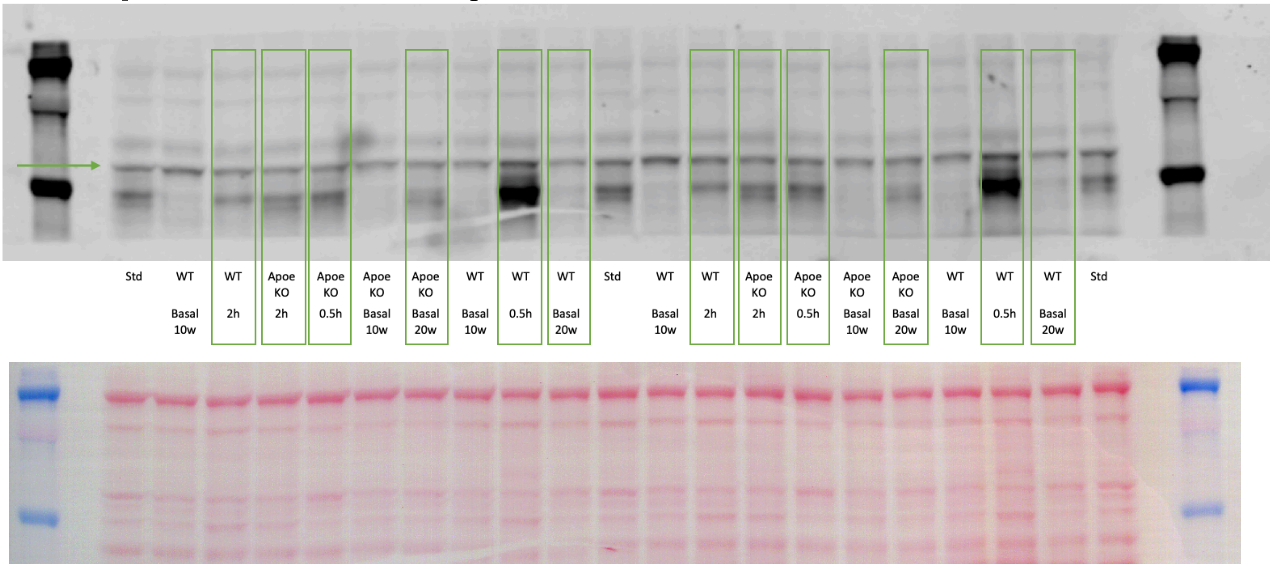

p-AKT1 (Thr308) – representative blots from figure 6b

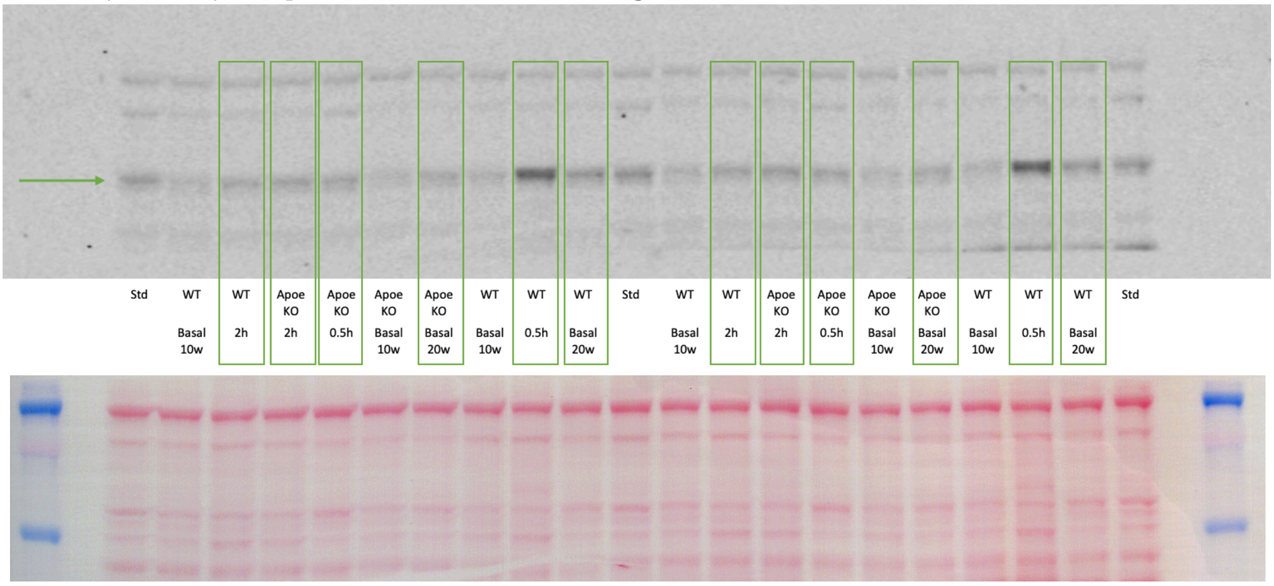

mTOR – representative blots from figure 6c

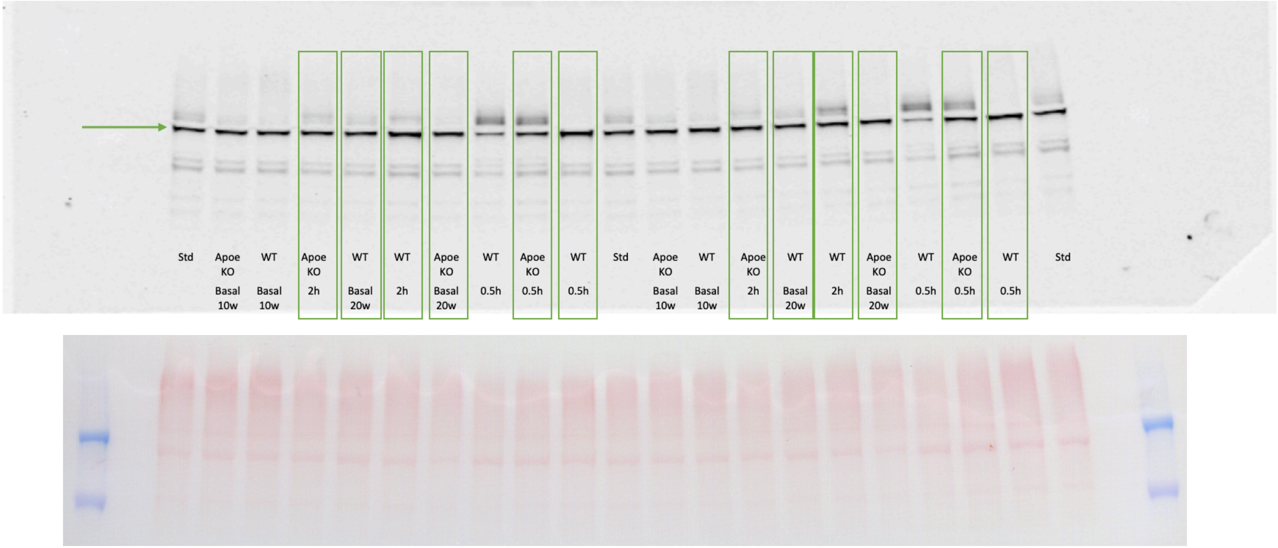

p-mTOR (Ser2448) – representative blots from figure 6d

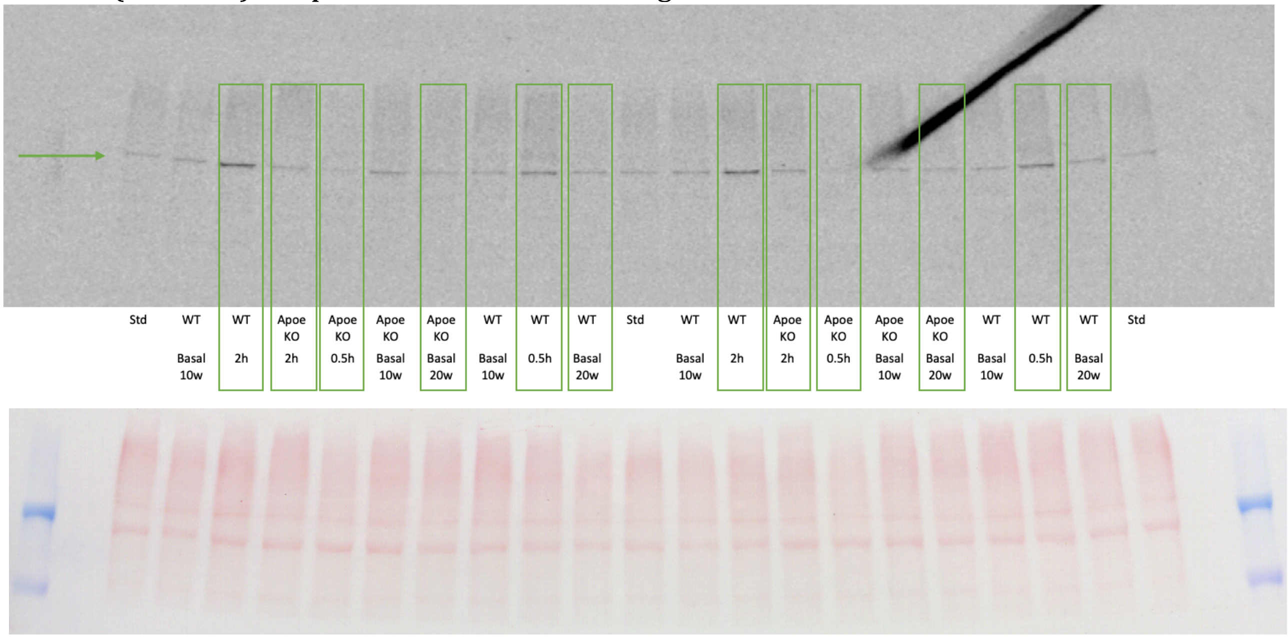

p-p70-S6K1 (Thr389) – representative blots from figure 6e

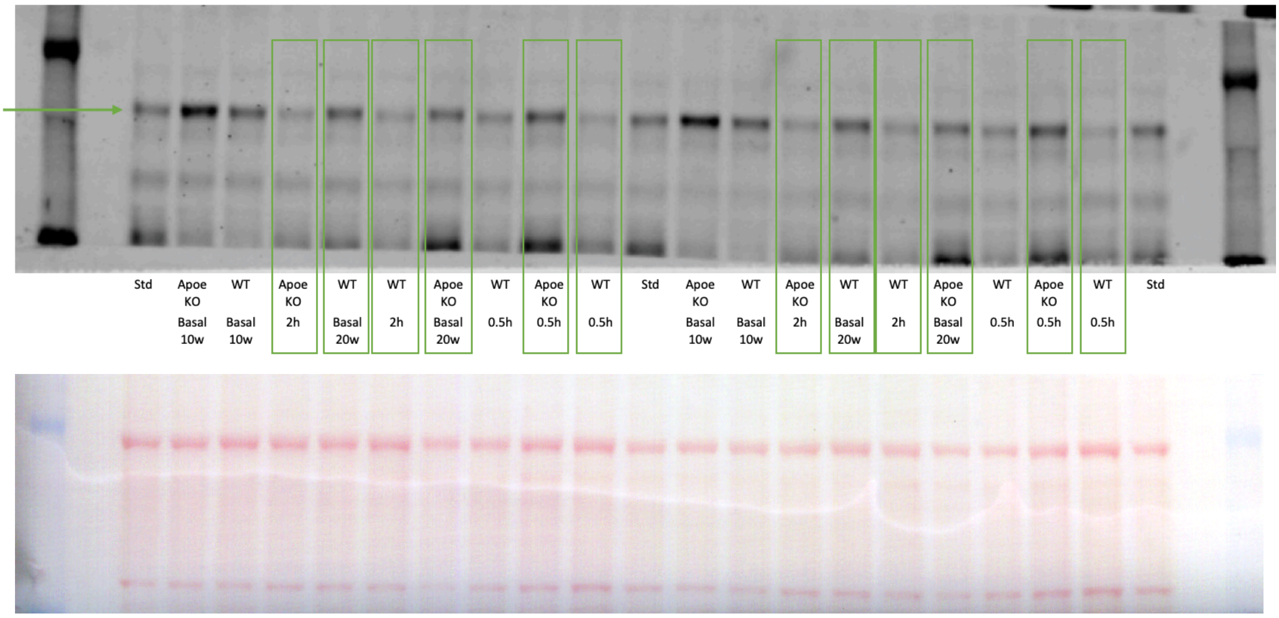

p-eEF2 (Thr56) – representative blots from figure 6f

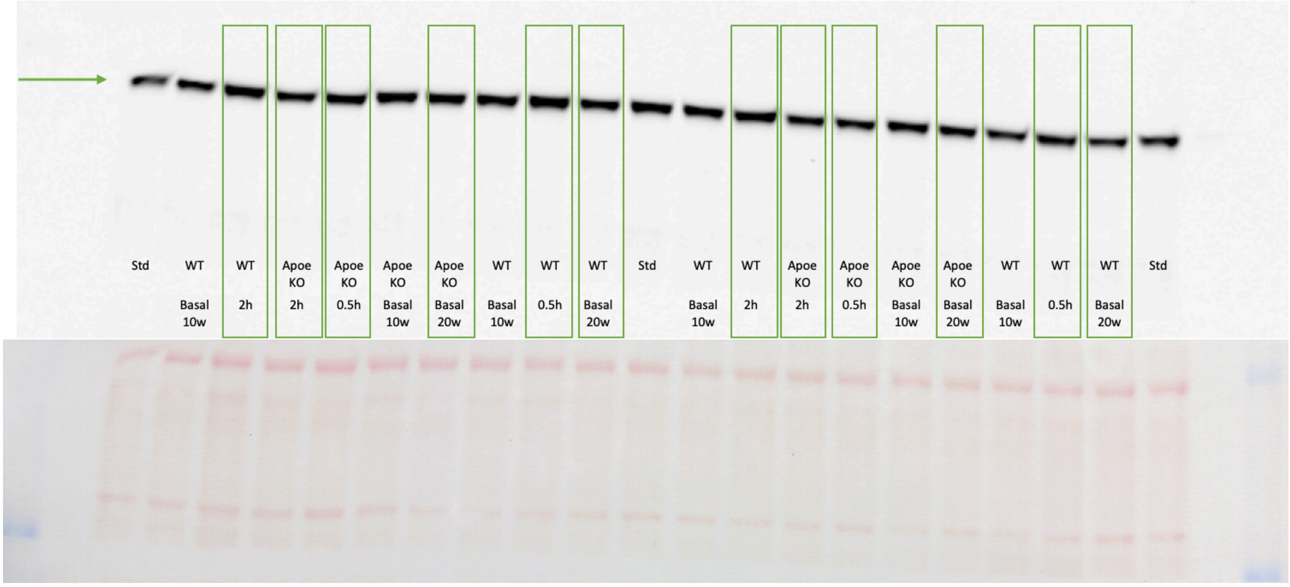

p-4E-BP1 (Thr37/46) – representative blots from figure 4g

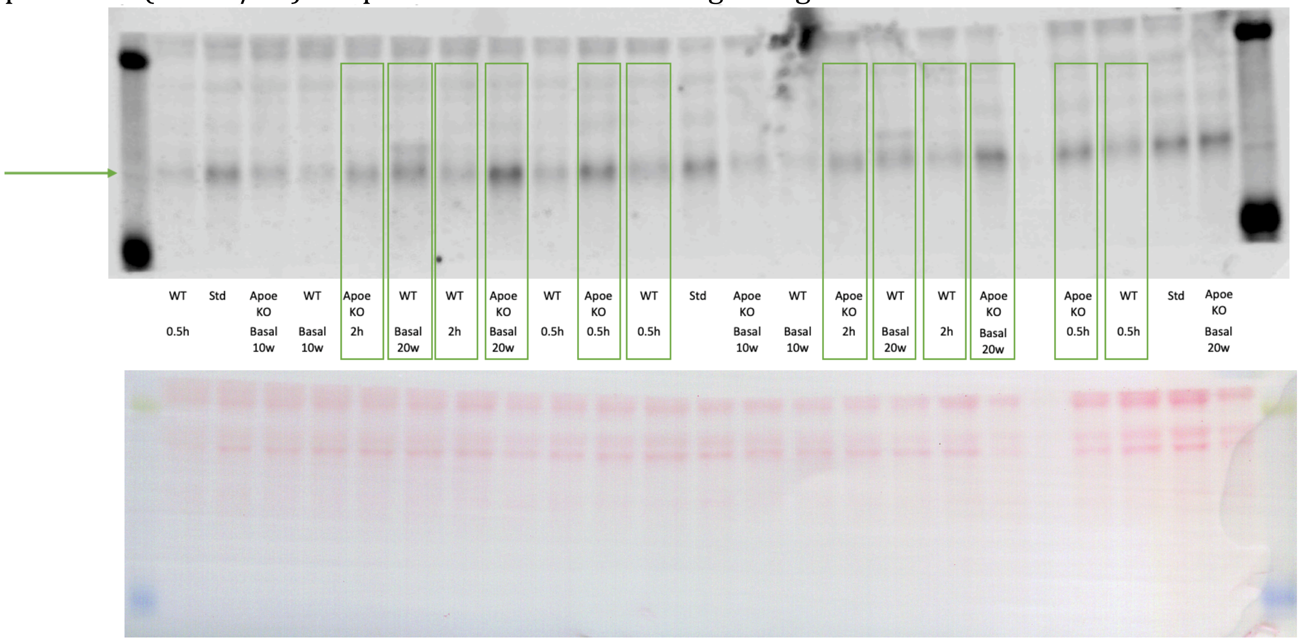

Supplemental figure S3: Representative blots of mTOR signaling targets with corresponding ponceau S stainings. Samples were loaded in a randomized order, repeated twice on the same gel. Genotype and time points are indicated on the figure. Green boxes indicate the bands that are shown as representative blots in figure 3 and 6, respectively.

SUPPLEMENTAL MATERIAL

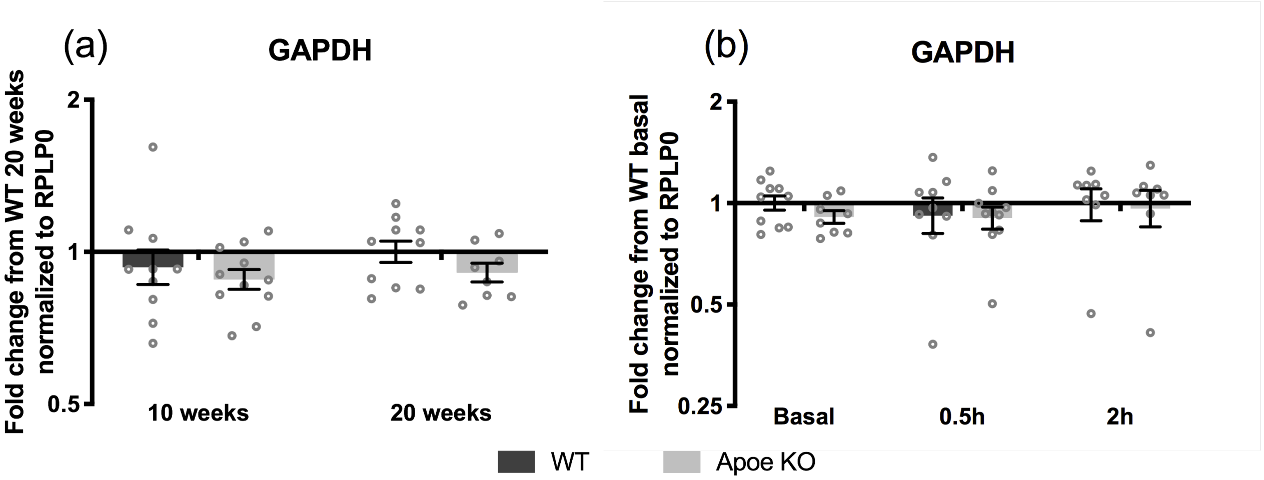

Supplemental figure S4: Glyceraldehyde 3-phosphate dehydrogenase (GAPDH) mRNA expression, in WT (dark bars) and Apoe KO (grey bars) at 10 weeks (WT n=10, Apoe KO n=11) and 20 weeks (WT n=10, Apoe KO n=8) basal, 0.5h (WT n=9, Apoe KO n=9), and 2h (WT n=8, Apoe KO n=8) after whey-protein ingestion.

As a control for normalization, GAPDH another often constitutively expressed mRNA was normalized to RPLP0. GAPDH normalized to RPLP0 showed no difference between any groups or changes over time, why we accept the normalization targets.

Data were normalized to RPLP0 and expressed as fold change from WT 20 weeks/basal and individual values are shown with bars of geometric means ± back-transformed SEM.

Supplemental table S1

Bacterial species significantly associated with both mice types and time analyzed with Bonferroni P G-test

A) WT vs. Apoe KO at 10 weeks

Species Level Summarized OTUs	Bonferroni P G test	WT 10 weeks mean	Apoe KO 10 weeks mean
k__Bacteria;p__Actinobacteria;c__Actinobacteria;o__Bifidobacteriales;f__Bifidobacteriaceae;g__Bifidobacterium;Other	2,68E-05	0,02%	0,08%
k__Bacteria;p__Actinobacteria;c__Coriobacteriia;o__Coriobacteriales;f__Coriobacteriaceae;g__Olsenella;Other	0,00E+00	0,06%	0,20%
k__Bacteria;p__Bacteroidetes;c__Bacteroidia;o__Bacteroidales;f__Bacteroidaceae;g__Bacteroides;s__Bacteroides acidifaciens	3,54E-02	0,00%	0,01%
k__Bacteria;p__Bacteroidetes;c__Bacteroidia;o__Bacteroidales;f__Muribaculaceae;g__Muribaculum;s__Muribaculum intestinale	1,00E-12	0,00%	0,05%
k__Bacteria;p__Bacteroidetes;c__Bacteroidia;o__Bacteroidales;f__Muribaculaceae;g__Muribaculum;s__PAC001076	1,88E-05	2,91%	2,49%
k__Bacteria;p__Bacteroidetes;c__Bacteroidia;o__Bacteroidales;f__Muribaculaceae;g__PAC001068;Other	5,58E-05	0,73%	0,96%
k__Bacteria;p__Bacteroidetes;c__Bacteroidia;o__Bacteroidales;f__Muribaculaceae;g__PAC001068;s__EF096118	2,83E-03	0,29%	0,42%
k__Bacteria;p__Bacteroidetes;c__Bacteroidia;o__Bacteroidales;f__Muribaculaceae;g__PAC001068;s__PAC001068	5,18E-03	0,86%	1,06%
k__Bacteria;p__Bacteroidetes;c__Bacteroidia;o__Bacteroidales;f__Muribaculaceae;g__PAC001068;s__PAC001736	2,68E-03	1,50%	1,77%
k__Bacteria;p__Bacteroidetes;c__Bacteroidia;o__Bacteroidales;f__Muribaculaceae;g__PAC001068;s__PAC001751	1,79E-09	0,94%	1,28%
k__Bacteria;p__Bacteroidetes;c__Bacteroidia;o__Bacteroidales;f__Muribaculaceae;g__PAC001112;Other	6,36E-06	0,31%	0,47%
k__Bacteria;p__Bacteroidetes;c__Bacteroidia;o__Bacteroidales;f__Muribaculaceae;g__PAC001127;s__PAC001482	8,43E-09	0,55%	0,81%
k__Bacteria;p__Bacteroidetes;c__Bacteroidia;o__Bacteroidales;f__Muribaculaceae;g__PAC001472;s__EU510580	4,02E-14	1,10%	0,73%
k__Bacteria;p__Bacteroidetes;c__Bacteroidia;o__Bacteroidales;f__Muribaculaceae;g__PAC001472;s__EU622749	8,04E-14	0,55%	0,30%
k__Bacteria;p__Bacteroidetes;c__Bacteroidia;o__Bacteroidales;f__Muribaculaceae;g__PAC001472;s__PAC001472	0,00E+00	2,48%	1,76%
k__Bacteria;p__Bacteroidetes;c__Bacteroidia;o__Bacteroidales;f__Muribaculaceae;g__PAC002400;Other	0,00E+00	0,00%	0,16%
k__Bacteria;p__Bacteroidetes;c__Bacteroidia;o__Bacteroidales;f__Muribaculaceae;g__PAC002400;s__EF096814	0,00E+00	0,00%	0,21%
k__Bacteria;p__Bacteroidetes;c__Bacteroidia;o__Bacteroidales;f__Muribaculaceae;g__PAC002448;s__AB606390	2,46E-02	0,00%	0,01%
k__Bacteria;p__Bacteroidetes;c__Bacteroidia;o__Bacteroidales;f__Rikenellaceae;g__Alistipes;s__PAC001471	2,92E-03	0,09%	0,04%
k__Bacteria;p__Bacteroidetes;c__Bacteroidia;o__Bacteroidales;f__Rikenellaceae;g__Alistipes;s__PAC002444	3,04E-02	0,08%	0,03%
k__Bacteria;p__Firmicutes;c__Bacilli;o__Lactobacillales;f__Lactobacillaceae;g__Lactobacillus;s__Lactobacillus animalis	0,00E+00	0,12%	0,76%
k__Bacteria;p__Firmicutes;c__Clostridia;o__Clostridiales;f__Clostridiaceae;g__Arthromitus;s__AP012202	5,17E-03	0,07%	0,13%
k__Bacteria;p__Firmicutes;c__Clostridia;o__Clostridiales;f__Lachnospiraceae;g__KE159538;s__PAC002428	0,00E+00	0,41%	0,02%
k__Bacteria;p__Firmicutes;c__Clostridia;o__Clostridiales;f__Lachnospiraceae;g__Marvinbryantia;s__PAC001753	1,90E-06	0,05%	0,01%
k__Bacteria;p__Firmicutes;c__Clostridia;o__Clostridiales;f__Lachnospiraceae;Other;Other	4,41E-08	4,67%	4,04%
k__Bacteria;p__Firmicutes;c__Clostridia;o__Clostridiales;f__Ruminococcaceae;g__Eubacterium23;Other	1,72E-02	0,16%	0,09%
k__Bacteria;p__Firmicutes;c__Clostridia;o__Clostridiales;f__Ruminococcaceae;g__Eubacterium8;Other	8,12E-06	0,13%	0,05%
k__Bacteria;p__Proteobacteria;c__Betaproteobacteria;o__Burkholderiales;f__Comamonadaceae;g__Comamonas;s__Comamonas testosteroni	1,57E-02	0,01%	0,00%
k__Bacteria;p__Proteobacteria;c__Betaproteobacteria;o__Burkholderiales;f__Sutterellaceae;g__Parasutterella;s__Parasutterella excrementihominis	1,29E-11	0,27%	0,12%
k__Bacteria;p__Proteobacteria;c__Deltaproteobacteria;o__Desulfovibrionales;f__Desulfovibrionaceae;g__LT706945;Other	4,02E-13	0,05%	0,00%
k__Bacteria;p__Verrucomicrobia;c__Verrucomicrobiae;o__Verrucomicrobiales;f__Akkermansiaceae;g__Akkermansia;Other	7,28E-04	0,24%	0,37%

B) WT vs. Apoe KO at 20 weeks

Species Level Summarized OTUs	Bonferroni P G test	WT 20 weeks mean	Apoe KO 20 weeks mean
k__Bacteria;p__Bacteroidetes;c__Bacteroidia;o__Bacteroidales;f__AC160630;g__PAC002482;s__EF603735	1,55E-02	0,07%	0,13%
k__Bacteria;p__Bacteroidetes;c__Bacteroidia;o__Bacteroidales;f__Bacteroidaceae;g__Bacteroides;Other	0,00E+00	0,06%	0,22%
k__Bacteria;p__Bacteroidetes;c__Bacteroidia;o__Bacteroidales;f__Bacteroidaceae;g__Bacteroides;s__Bacteroides caecimuris	6,49E-03	0,02%	0,06%
k__Bacteria;p__Bacteroidetes;c__Bacteroidia;o__Bacteroidales;f__Bacteroidaceae;g__Bacteroides;s__Bacteroides uniformis	1,71E-05	0,03%	0,09%
k__Bacteria;p__Bacteroidetes;c__Bacteroidia;o__Bacteroidales;f__Bacteroidaceae;g__Bacteroides;s__HM124113	2,75E-05	0,02%	0,07%
k__Bacteria;p__Bacteroidetes;c__Bacteroidia;o__Bacteroidales;f__Muribaculaceae;g__HM124200;s__EF096297	1,99E-03	1,42%	1,18%
k__Bacteria;p__Bacteroidetes;c__Bacteroidia;o__Bacteroidales;f__Muribaculaceae;g__Muribaculum;Other	2,77E-11	0,20%	0,07%
k__Bacteria;p__Bacteroidetes;c__Bacteroidia;o__Bacteroidales;f__Muribaculaceae;g__Muribaculum;s__PAC001076	0,00E+00	2,18%	1,23%
k__Bacteria;p__Bacteroidetes;c__Bacteroidia;o__Bacteroidales;f__Muribaculaceae;g__Muribaculum;s__PAC001735	7,03E-12	2,22%	1,72%
k__Bacteria;p__Bacteroidetes;c__Bacteroidia;o__Bacteroidales;f__Muribaculaceae;g__PAC000186;s__PAC001128	1,05E-06	0,21%	0,35%
k__Bacteria;p__Bacteroidetes;c__Bacteroidia;o__Bacteroidales;f__Muribaculaceae;g__PAC000186;s__PAC001483	1,78E-10	13,14%	14,40%
k__Bacteria;p__Bacteroidetes;c__Bacteroidia;o__Bacteroidales;f__Muribaculaceae;g__PAC000186;s__PAC001672	3,24E-04	1,10%	1,35%
k__Bacteria;p__Bacteroidetes;c__Bacteroidia;o__Bacteroidales;f__Muribaculaceae;g__PAC000198;Other	5,00E-07	0,44%	0,27%
k__Bacteria;p__Bacteroidetes;c__Bacteroidia;o__Bacteroidales;f__Muribaculaceae;g__PAC000198;s__PAC001513	2,09E-06	0,57%	0,38%
k__Bacteria;p__Bacteroidetes;c__Bacteroidia;o__Bacteroidales;f__Muribaculaceae;g__PAC000198;s__PAC002296	0,00E+00	2,49%	1,60%
k__Bacteria;p__Bacteroidetes;c__Bacteroidia;o__Bacteroidales;f__Muribaculaceae;g__PAC001063;s__PAC001063	3,24E-09	0,46%	0,27%
k__Bacteria;p__Bacteroidetes;c__Bacteroidia;o__Bacteroidales;f__Muribaculaceae;g__PAC001068;s__EF097112	0,00E+00	2,88%	3,91%
k__Bacteria;p__Bacteroidetes;c__Bacteroidia;o__Bacteroidales;f__Muribaculaceae;g__PAC001068;s__PAC001068	4,04E-04	0,82%	0,62%
k__Bacteria;p__Bacteroidetes;c__Bacteroidia;o__Bacteroidales;f__Muribaculaceae;g__PAC001068;s__PAC001736	1,35E-05	1,44%	1,15%
k__Bacteria;p__Bacteroidetes;c__Bacteroidia;o__Bacteroidales;f__Muribaculaceae;g__PAC001068;s__PAC001751	0,00E+00	0,33%	0,74%
k__Bacteria;p__Bacteroidetes;c__Bacteroidia;o__Bacteroidales;f__Muribaculaceae;g__PAC001127;s__PAC001482	3,46E-11	0,74%	0,47%
k__Bacteria;p__Bacteroidetes;c__Bacteroidia;o__Bacteroidales;f__Muribaculaceae;g__PAC001472;s__PAC001472	0,00E+00	2,79%	1,42%
k__Bacteria;p__Bacteroidetes;c__Bacteroidia;o__Bacteroidales;f__Muribaculaceae;g__PAC001512;Other	1,17E-02	0,00%	0,02%
k__Bacteria;p__Bacteroidetes;c__Bacteroidia;o__Bacteroidales;f__Muribaculaceae;g__PAC001512;s__PAC001512	9,10E-04	0,00%	0,03%
k__Bacteria;p__Bacteroidetes;c__Bacteroidia;o__Bacteroidales;f__Muribaculaceae;g__PAC001765;s__PAC001765	8,39E-05	0,47%	0,31%
k__Bacteria;p__Bacteroidetes;c__Bacteroidia;o__Bacteroidales;f__Porphyromonadaceae;g__Parabacteroides;Other	7,18E-05	0,02%	0,00%
k__Bacteria;p__Bacteroidetes;c__Bacteroidia;o__Bacteroidales;f__Prevotellaceae;g__Prevotella;Other	1,09E-12	0,04%	0,16%
k__Bacteria;p__Bacteroidetes;c__Bacteroidia;o__Bacteroidales;f__Prevotellaceae;g__Prevotella;s__PAC002481	0,00E+00	0,12%	0,39%
k__Bacteria;p__Bacteroidetes;c__Bacteroidia;o__Bacteroidales;f__Rikenellaceae;g__Alistipes;Other	2,50E-03	0,15%	0,24%
k__Bacteria;p__Bacteroidetes;c__Bacteroidia;o__Bacteroidales;f__Rikenellaceae;g__Alistipes;s__PAC002444	1,16E-07	0,04%	0,00%
k__Bacteria;p__Bacteroidetes;c__Bacteroidia;o__Bacteroidales;Other;Other;Other	1,64E-08	0,28%	0,47%
k__Bacteria;p__Firmicutes;c__Bacilli;o__Lactobacillales;f__Lactobacillaceae;g__Lactobacillus;s__Lactobacillus animalis	0,00E+00	0,16%	2,10%
k__Bacteria;p__Firmicutes;c__Bacilli;o__Lactobacillales;f__Lactobacillaceae;g__Lactobacillus;s__Lactobacillus johnsonii	5,85E-05	0,13%	0,24%
k__Bacteria;p__Firmicutes;c__Clostridia;o__Clostridiales;f__Clostridiaceae;g__Arthromitus;s__AP012202	1,30E-11	0,13%	0,29%
k__Bacteria;p__Firmicutes;c__Clostridia;o__Clostridiales;f__Lachnospiraceae;g__KE159538;s__PAC001708	1,14E-09	0,05%	0,00%
k__Bacteria;p__Firmicutes;c__Clostridia;o__Clostridiales;f__Lachnospiraceae;g__KE159538;s__PAC001770	9,65E-06	0,13%	0,05%
k__Bacteria;p__Firmicutes;c__Clostridia;o__Clostridiales;f__Lachnospiraceae;g__KE159538;s__PAC002428	2,18E-05	0,05%	0,01%
k__Bacteria;p__Firmicutes;c__Clostridia;o__Clostridiales;f__Lachnospiraceae;g__Marvinbryantia;s__PAC001753	4,90E-02	0,05%	0,02%
k__Bacteria;p__Firmicutes;c__Clostridia;o__Clostridiales;f__Lachnospiraceae;g__PAC002368;s__PAC002368	7,58E-03	0,02%	0,00%
k__Bacteria;p__Firmicutes;c__Clostridia;o__Clostridiales;f__Lachnospiraceae;Other;Other	0,00E+00	4,50%	3,42%
k__Bacteria;p__Firmicutes;c__Clostridia;o__Clostridiales;f__Ruminococcaceae;g__Eubacterium23;Other	2,75E-03	0,19%	0,11%
k__Bacteria;p__Firmicutes;c__Clostridia;o__Clostridiales;f__Ruminococcaceae;g__PAC000661;s__PAC000661	1,12E-04	0,05%	0,01%
k__Bacteria;p__Firmicutes;c__Clostridia;o__Clostridiales;f__Ruminococcaceae;g__PAC000661;s__PAC002109	1,19E-03	0,00%	0,03%
k__Bacteria;p__Firmicutes;c__Clostridia;o__Clostridiales;f__Ruminococcaceae;g__PAC000683;Other	3,22E-05	0,01%	0,06%
k__Bacteria;p__Firmicutes;c__Clostridia;o__Clostridiales;f__Ruminococcaceae;g__Ruminococcus;Other	3,69E-10	0,08%	0,01%
k__Bacteria;p__Firmicutes;c__Clostridia;o__Clostridiales;f__Ruminococcaceae;g__Ruminococcus;s__HM124121	2,16E-07	0,10%	0,03%
k__Bacteria;p__Firmicutes;c__Clostridia;o__Clostridiales;f__Ruminococcaceae;Other;Other	1,34E-03	0,16%	0,08%
k__Bacteria;p__Proteobacteria;c__Deltaproteobacteria;o__Desulfovibrionales;f__Desulfovibrionaceae;g__LT706945;Other	0,00E+00	0,13%	0,00%
k__Bacteria;p__Verrucomicrobia;c__Verrucomicrobiae;o__Verrucomicrobiales;f__Akkermansiaceae;g__Akkermansia;Other	0,00E+00	0,02%	1,78%

C) WT 10 vs. 20 weeks

Species Level Summarized OTUs	Bonferroni P G test	WT 10 weeks mean	WT 20 weeks mean
k__Bacteria;p__Actinobacteria;c__Actinobacteria;o__Bifidobacteriales;f__Bifidobacteriaceae;g__Bifidobacterium;Other	3,02E-05	0,02%	0,00%
k__Bacteria;p__Actinobacteria;c__Coriobacteriia;o__Coriobacteriales;f__Coriobacteriaceae;g__Olsenella;Other	2,14E-08	0,06%	0,01%
k__Bacteria;p__Bacteroidetes;c__Bacteroidia;o__Bacteroidales;f__Muribaculaceae;g__HM124200;s__EF096297	2,01E-13	1,00%	1,42%
k__Bacteria;p__Bacteroidetes;c__Bacteroidia;o__Bacteroidales;f__Muribaculaceae;g__Muribaculum;s__PAC001076	0,00E+00	2,91%	2,18%
k__Bacteria;p__Bacteroidetes;c__Bacteroidia;o__Bacteroidales;f__Muribaculaceae;g__Muribaculum;s__PAC001735	0,00E+00	1,57%	2,22%
k__Bacteria;p__Bacteroidetes;c__Bacteroidia;o__Bacteroidales;f__Muribaculaceae;g__PAC000198;s__PAC001075	2,97E-02	0,01%	0,00%
k__Bacteria;p__Bacteroidetes;c__Bacteroidia;o__Bacteroidales;f__Muribaculaceae;g__PAC000198;s__PAC001513	4,51E-03	0,43%	0,57%
k__Bacteria;p__Bacteroidetes;c__Bacteroidia;o__Bacteroidales;f__Muribaculaceae;g__PAC000198;s__PAC002296	0,00E+00	1,66%	2,49%
k__Bacteria;p__Bacteroidetes;c__Bacteroidia;o__Bacteroidales;f__Muribaculaceae;g__PAC001063;s__PAC001063	2,11E-06	0,29%	0,46%
k__Bacteria;p__Bacteroidetes;c__Bacteroidia;o__Bacteroidales;f__Muribaculaceae;g__PAC001068;Other	0,00E+00	0,73%	0,24%
k__Bacteria;p__Bacteroidetes;c__Bacteroidia;o__Bacteroidales;f__Muribaculaceae;g__PAC001068;s__EF096118	0,00E+00	0,29%	0,00%
k__Bacteria;p__Bacteroidetes;c__Bacteroidia;o__Bacteroidales;f__Muribaculaceae;g__PAC001068;s__EF097112	2,83E-06	3,36%	2,88%
k__Bacteria;p__Bacteroidetes;c__Bacteroidia;o__Bacteroidales;f__Muribaculaceae;g__PAC001068;s__PAC001070	0,00E+00	0,08%	0,00%
k__Bacteria;p__Bacteroidetes;c__Bacteroidia;o__Bacteroidales;f__Muribaculaceae;g__PAC001068;s__PAC001751	0,00E+00	0,94%	0,33%
k__Bacteria;p__Bacteroidetes;c__Bacteroidia;o__Bacteroidales;f__Muribaculaceae;g__PAC001112;Other	1,20E-03	0,31%	0,44%
k__Bacteria;p__Bacteroidetes;c__Bacteroidia;o__Bacteroidales;f__Muribaculaceae;g__PAC001127;s__PAC001482	4,34E-04	0,55%	0,74%
k__Bacteria;p__Bacteroidetes;c__Bacteroidia;o__Bacteroidales;f__Muribaculaceae;g__PAC001472;Other	1,83E-05	0,17%	0,08%
k__Bacteria;p__Bacteroidetes;c__Bacteroidia;o__Bacteroidales;f__Muribaculaceae;g__PAC001472;s__EU510580	0,00E+00	1,10%	0,71%
k__Bacteria;p__Bacteroidetes;c__Bacteroidia;o__Bacteroidales;f__Muribaculaceae;g__PAC001472;s__EU622749	0,00E+00	0,55%	0,20%
k__Bacteria;p__Bacteroidetes;c__Bacteroidia;o__Bacteroidales;f__Muribaculaceae;g__PAC001472;s__PAC001472	2,03E-02	2,48%	2,79%
k__Bacteria;p__Bacteroidetes;c__Bacteroidia;o__Bacteroidales;f__Muribaculaceae;g__PAC001765;s__PAC001765	4,71E-07	0,29%	0,47%
k__Bacteria;p__Bacteroidetes;c__Bacteroidia;o__Bacteroidales;f__Muribaculaceae;Other;Other	0,00E+00	46,99%	51,61%
k__Bacteria;p__Firmicutes;c__Bacilli;o__Lactobacillales;f__Lactobacillaceae;g__Lactobacillus;Other	2,09E-04	0,01%	0,04%
k__Bacteria;p__Firmicutes;c__Bacilli;o__Lactobacillales;f__Lactobacillaceae;g__Lactobacillus;s__Lactobacillus intestinalis	1,59E-05	0,00%	0,03%
k__Bacteria;p__Firmicutes;c__Bacilli;o__Lactobacillales;f__Lactobacillaceae;g__Lactobacillus;s__Lactobacillus johnsonii	2,34E-07	0,05%	0,13%
k__Bacteria;p__Firmicutes;c__Bacilli;o__Lactobacillales;f__Lactobacillaceae;g__Lactobacillus;s__Lactobacillus vaginalis	6,56E-10	0,01%	0,09%
k__Bacteria;p__Firmicutes;c__Clostridia;o__Clostridiales;f__Clostridiaceae;g__Arthromitus;s__AP012202	5,16E-03	0,07%	0,13%
k__Bacteria;p__Firmicutes;c__Clostridia;o__Clostridiales;f__Lachnospiraceae;g__KE159538;s__PAC001708	1,35E-03	0,01%	0,05%
k__Bacteria;p__Firmicutes;c__Clostridia;o__Clostridiales;f__Lachnospiraceae;g__KE159538;s__PAC001770	0,00E+00	0,02%	0,13%
k__Bacteria;p__Firmicutes;c__Clostridia;o__Clostridiales;f__Lachnospiraceae;g__KE159538;s__PAC002428	0,00E+00	0,41%	0,05%
k__Bacteria;p__Firmicutes;c__Clostridia;o__Clostridiales;f__Lachnospiraceae;g__KE159628;s__KE159628	2,73E-03	0,01%	0,05%
k__Bacteria;p__Firmicutes;c__Clostridia;o__Clostridiales;f__Lachnospiraceae;g__PAC000664;Other	1,22E-06	0,53%	0,35%
k__Bacteria;p__Firmicutes;c__Clostridia;o__Clostridiales;f__Lachnospiraceae;g__PAC000664;s__AB606242	1,09E-07	0,01%	0,06%
k__Bacteria;p__Firmicutes;c__Clostridia;o__Clostridiales;f__Peptococcaceae;g__PAC001500;s__PAC001500	4,80E-02	0,01%	0,03%
k__Bacteria;p__Firmicutes;c__Clostridia;o__Clostridiales;f__Ruminococcaceae;g__PAC000661;Other	2,03E-02	0,13%	0,20%
k__Bacteria;p__Firmicutes;c__Clostridia;o__Clostridiales;f__Ruminococcaceae;g__PAC000661;s__PAC000661	1,77E-08	0,00%	0,05%
k__Bacteria;p__Firmicutes;c__Clostridia;o__Clostridiales;f__Ruminococcaceae;g__PAC000661;s__PAC002109	8,99E-04	0,03%	0,00%
k__Bacteria;p__Firmicutes;c__Clostridia;o__Clostridiales;f__Ruminococcaceae;g__Ruminococcus;s__HM124121	3,31E-03	0,04%	0,10%
k__Bacteria;p__Firmicutes;c__Erysipelotrichi;o__Erysipelotrichales;f__Erysipelotrichaceae;g__Coprobacillus;s__PAC002396	1,66E-06	0,00%	0,03%
k__Bacteria;p__Firmicutes;c__Erysipelotrichi;o__Erysipelotrichales;f__Erysipelotrichaceae;g__Faecalibaculum;s__Faecalibaculum rodentium	0,00E+00	4,59%	0,11%
k__Bacteria;p__Firmicutes;Other;Other;Other;Other;Other	2,95E-03	0,03%	0,00%
k__Bacteria;p__Proteobacteria;c__Betaproteobacteria;o__Burkholderiales;f__Comamonadaceae;g__Comamonas;s__Comamonas testosteroni	1,57E-02	0,01%	0,00%
k__Bacteria;p__Proteobacteria;c__Betaproteobacteria;o__Burkholderiales;f__Sutterellaceae;g__Parasutterella;s__Parasutterella excrementihominis	6,03E-13	0,27%	0,11%
k__Bacteria;p__Proteobacteria;c__Deltaproteobacteria;o__Desulfovibrionales;f__Desulfovibrionaceae;g__LT706945;Other	3,67E-06	0,05%	0,13%
k__Bacteria;p__Verrucomicrobia;c__Verrucomicrobiae;o__Verrucomicrobiales;f__Akkermansiaceae;g__Akkermansia;Other	0,00E+00	0,24%	0,02%

D) Apoe KO 10 vs. 20 weeks

Species Level Summarized OTUs	Bonferroni P G test	Apoe KO 10 weeks mean	Apoe KO 20 weeks mean
k__Bacteria;p__Actinobacteria;c__Actinobacteria;o__Bifidobacteriales;f__Bifidobacteriaceae;g__Bifidobacterium;Other	0,00E+00	0,08%	0,00%
k__Bacteria;p__Actinobacteria;c__Coriobacteriia;o__Coriobacteriales;f__Coriobacteriaceae;g__Olsenella;Other	0,00E+00	0,20%	0,01%
k__Bacteria;p__Bacteroidetes;c__Bacteroidia;o__Bacteroidales;f__AC160630;g__PAC002482;s__EF603735	1,66E-08	0,04%	0,13%
k__Bacteria;p__Bacteroidetes;c__Bacteroidia;o__Bacteroidales;f__Bacteroidaceae;g__Bacteroides;Other	0,00E+00	0,07%	0,22%
k__Bacteria;p__Bacteroidetes;c__Bacteroidia;o__Bacteroidales;f__Bacteroidaceae;g__Bacteroides;s__Bacteroides acidifaciens	2,76E-02	0,01%	0,00%
k__Bacteria;p__Bacteroidetes;c__Bacteroidia;o__Bacteroidales;f__Bacteroidaceae;g__Bacteroides;s__Bacteroides caecimuris	2,94E-02	0,02%	0,06%
k__Bacteria;p__Bacteroidetes;c__Bacteroidia;o__Bacteroidales;f__Bacteroidaceae;g__Bacteroides;s__Bacteroides uniformis	1,64E-09	0,02%	0,09%
k__Bacteria;p__Bacteroidetes;c__Bacteroidia;o__Bacteroidales;f__Bacteroidaceae;g__Bacteroides;s__HM124113	5,32E-08	0,01%	0,07%
k__Bacteria;p__Bacteroidetes;c__Bacteroidia;o__Bacteroidales;f__Muribaculaceae;g__Muribaculum;s__Muribaculum intestinale	4,82E-13	0,05%	0,00%
k__Bacteria;p__Bacteroidetes;c__Bacteroidia;o__Bacteroidales;f__Muribaculaceae;g__Muribaculum;s__PAC001076	0,00E+00	2,49%	1,23%
k__Bacteria;p__Bacteroidetes;c__Bacteroidia;o__Bacteroidales;f__Muribaculaceae;g__PAC000186;s__PAC001128	2,03E-07	0,20%	0,35%
k__Bacteria;p__Bacteroidetes;c__Bacteroidia;o__Bacteroidales;f__Muribaculaceae;g__PAC000186;s__PAC001483	1,85E-09	13,19%	14,40%
k__Bacteria;p__Bacteroidetes;c__Bacteroidia;o__Bacteroidales;f__Muribaculaceae;g__PAC000186;s__PAC001672	1,13E-03	1,11%	1,35%
k__Bacteria;p__Bacteroidetes;c__Bacteroidia;o__Bacteroidales;f__Muribaculaceae;g__PAC001068;Other	0,00E+00	0,96%	0,30%
k__Bacteria;p__Bacteroidetes;c__Bacteroidia;o__Bacteroidales;f__Muribaculaceae;g__PAC001068;s__EF096118	0,00E+00	0,42%	0,00%
k__Bacteria;p__Bacteroidetes;c__Bacteroidia;o__Bacteroidales;f__Muribaculaceae;g__PAC001068;s__EF097112	0,00E+00	3,06%	3,91%
k__Bacteria;p__Bacteroidetes;c__Bacteroidia;o__Bacteroidales;f__Muribaculaceae;g__PAC001068;s__PAC001068	0,00E+00	1,06%	0,62%
k__Bacteria;p__Bacteroidetes;c__Bacteroidia;o__Bacteroidales;f__Muribaculaceae;g__PAC001068;s__PAC001070	0,00E+00	0,12%	0,00%
k__Bacteria;p__Bacteroidetes;c__Bacteroidia;o__Bacteroidales;f__Muribaculaceae;g__PAC001068;s__PAC001736	0,00E+00	1,77%	1,15%
k__Bacteria;p__Bacteroidetes;c__Bacteroidia;o__Bacteroidales;f__Muribaculaceae;g__PAC001068;s__PAC001751	0,00E+00	1,28%	0,74%
k__Bacteria;p__Bacteroidetes;c__Bacteroidia;o__Bacteroidales;f__Muribaculaceae;g__PAC001112;Other	4,52E-02	0,47%	0,35%
k__Bacteria;p__Bacteroidetes;c__Bacteroidia;o__Bacteroidales;f__Muribaculaceae;g__PAC001127;s__PAC001482	0,00E+00	0,81%	0,47%
k__Bacteria;p__Bacteroidetes;c__Bacteroidia;o__Bacteroidales;f__Muribaculaceae;g__PAC001472;s__EU622749	1,59E-03	0,30%	0,19%
k__Bacteria;p__Bacteroidetes;c__Bacteroidia;o__Bacteroidales;f__Muribaculaceae;g__PAC001472;s__PAC001472	3,81E-06	1,76%	1,42%
k__Bacteria;p__Bacteroidetes;c__Bacteroidia;o__Bacteroidales;f__Muribaculaceae;g__PAC002400;Other	0,00E+00	0,16%	0,00%
k__Bacteria;p__Bacteroidetes;c__Bacteroidia;o__Bacteroidales;f__Muribaculaceae;g__PAC002400;s__EF096814	0,00E+00	0,21%	0,00%
k__Bacteria;p__Bacteroidetes;c__Bacteroidia;o__Bacteroidales;f__Muribaculaceae;g__PAC002448;s__AB606390	2,46E-02	0,01%	0,00%
k__Bacteria;p__Bacteroidetes;c__Bacteroidia;o__Bacteroidales;f__Muribaculaceae;Other;Other	0,00E+00	47,46%	50,68%
k__Bacteria;p__Bacteroidetes;c__Bacteroidia;o__Bacteroidales;f__Prevotellaceae;g__Prevotella;s__PAC002481	1,26E-05	0,25%	0,39%
k__Bacteria;p__Bacteroidetes;c__Bacteroidia;o__Bacteroidales;f__Rikenellaceae;g__Alistipes;s__PAC001061	5,11E-09	0,03%	0,11%
k__Bacteria;p__Bacteroidetes;c__Bacteroidia;o__Bacteroidales;f__Rikenellaceae;g__Alistipes;s__PAC001471	4,43E-02	0,04%	0,08%
k__Bacteria;p__Bacteroidetes;c__Bacteroidia;o__Bacteroidales;f__Rikenellaceae;g__Alistipes;s__PAC002444	1,12E-06	0,03%	0,00%
k__Bacteria;p__Bacteroidetes;c__Bacteroidia;o__Bacteroidales;Other;Other;Other	6,52E-05	0,32%	0,47%
k__Bacteria;p__Firmicutes;c__Bacilli;o__Lactobacillales;f__Lactobacillaceae;g__Lactobacillus;Other	1,60E-08	0,01%	0,05%
k__Bacteria;p__Firmicutes;c__Bacilli;o__Lactobacillales;f__Lactobacillaceae;g__Lactobacillus;s__Lactobacillus animalis	0,00E+00	0,76%	2,10%
k__Bacteria;p__Firmicutes;c__Bacilli;o__Lactobacillales;f__Lactobacillaceae;g__Lactobacillus;s__Lactobacillus intestinalis	1,04E-05	0,00%	0,03%
k__Bacteria;p__Firmicutes;c__Bacilli;o__Lactobacillales;f__Lactobacillaceae;g__Lactobacillus;s__Lactobacillus johnsonii	0,00E+00	0,06%	0,24%
k__Bacteria;p__Firmicutes;c__Bacilli;o__Lactobacillales;f__Lactobacillaceae;g__Lactobacillus;s__Lactobacillus vaginalis	0,00E+00	0,02%	0,12%
k__Bacteria;p__Firmicutes;c__Clostridia;o__Clostridiales;f__Clostridiaceae;g__Arthromitus;s__AP012202	1,30E-11	0,13%	0,29%
k__Bacteria;p__Firmicutes;c__Clostridia;o__Clostridiales;f__Lachnospiraceae;g__KE159538;s__PAC001770	5,82E-08	0,00%	0,05%
k__Bacteria;p__Firmicutes;c__Clostridia;o__Clostridiales;f__Lachnospiraceae;g__PAC000664;Other	4,07E-03	0,47%	0,34%
k__Bacteria;p__Firmicutes;c__Clostridia;o__Clostridiales;f__Lachnospiraceae;g__PAC000664;s__AB606242	1,02E-08	0,00%	0,05%
k__Bacteria;p__Firmicutes;c__Clostridia;o__Clostridiales;f__Lachnospiraceae;Other;Other	2,93E-09	4,04%	3,42%
k__Bacteria;p__Firmicutes;c__Clostridia;o__Clostridiales;f__Peptococcaceae;g__PAC001500;s__PAC001500	4,40E-05	0,00%	0,03%
k__Bacteria;p__Firmicutes;c__Clostridia;o__Clostridiales;f__Ruminococcaceae;g__PAC000661;Other	6,91E-06	0,16%	0,28%
k__Bacteria;p__Firmicutes;c__Clostridia;o__Clostridiales;f__Ruminococcaceae;g__PAC000683;Other	4,93E-08	0,01%	0,06%
k__Bacteria;p__Firmicutes;c__Clostridia;o__Clostridiales;f__Ruminococcaceae;g__PAC001402;s__PAC001546	3,76E-03	0,01%	0,04%
k__Bacteria;p__Firmicutes;c__Clostridia;o__Clostridiales;f__Ruminococcaceae;g__Pseudoflavonifractor;s__FJ879507	8,73E-11	0,00%	0,05%
k__Bacteria;p__Firmicutes;c__Erysipelotrichi;o__Erysipelotrichales;f__Erysipelotrichaceae;g__Faecalibaculum;s__Faecalibaculum rodentium	0,00E+00	4,44%	0,10%
k__Bacteria;p__Firmicutes;Other;Other;Other;Other;Other	2,08E-02	0,03%	0,00%
k__Bacteria;p__Verrucomicrobia;c__Verrucomicrobiae;o__Verrucomicrobiales;f__Akkermansiaceae;g__Akkermansia;Other	0,00E+00	0,37%	1,78%
